# CO_2_‐sensitive K^+^ channel traffic affects stomata and whole‐plant water use

**DOI:** 10.1111/jipb.70269

**Published:** 2026-04-22

**Authors:** Zhiyi Yu, Sakharam Waghmare, Sahar Farami, Michael R. Blatt, Rucha Karnik

**Affiliations:** ^1^ The School of Molecular Biosciences, College of Medical, Veterinary and Life Sciences University of Glasgow Glasgow G12 8QQ UK; ^2^ Laboratory of Plant Physiology and Biophysics University of Glasgow Glasgow G12 8QQ UK

**Keywords:** CO_2_‐sensing, K^+^ channel, membrane traffic, SNARE, stomata, transport

## Abstract

Stomata are pores at the leaf surface that facilitate CO_2_ entry for photosynthesis while controlling transpirational water loss. Stomatal movements are governed by reversible changes in turgor and cell volume, driven by the transport of osmotic solute across the membrane of guard cells surrounding the pore. Membrane transport depends on the activities of transporters and ion channels present at the membrane and also on their abundance, determined by vesicle traffic. Although much is known about how pumps and ion channels in the membrane are regulated, this is not the case for vesicle trafficking and certainly not in relation to CO_2_. Here, we report on experiments following the traffic of the K^+^ channel KAT1 that is important for K^+^ uptake during stomatal opening. We found that elevated CO_2_ triggers changes in channel mobility within the plane of the plasma membrane and its internalization therefrom. CO_2_‐sensitive KAT1 traffic depends on SYP121, a vesicle‐trafficking (SNARE) protein previously associated with water stress and abscisic acid (ABA) that also binds the K^+^ channel to promote its activation. The CO_2_‐sensitive pathway parallels vesicle traffic evoked by ABA, but it also shows important differences, with KAT1–SYP121 binding sensitive to CO_2_. We show that stomatal response to changes in CO_2_ levels is slowed in the *syp121* null mutant, affecting shoot growth and whole‐plant water‐use efficiency. Thus, we uncover a new target for CO_2_ regulation in vesicle traffic and a novel perspective on plant responses to climate change.

## Graphical abstract

CO_2_‐responsive regulation of membrane trafficking influences the density of the potassium ion (K^+^) channel KAT1 at the plasma membrane. CO2‐sensitive traffic, dependent on trafficking by the SNARE SYP121, impacts stomatal gas exchange and plant growth.

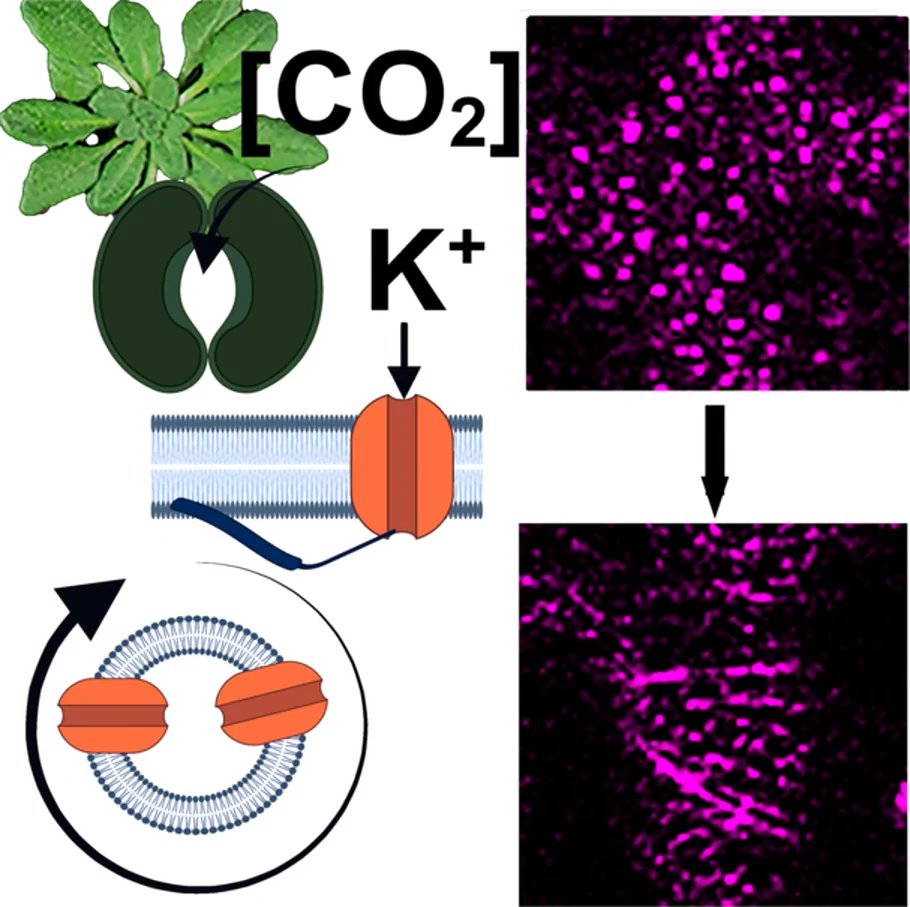

## INTRODUCTION

Increasing atmospheric carbon dioxide (CO_2_) and associated effects on the global water cycle are widely recognized as central to environmental sustainability and future food security (UNFCCC‐COP25, 2019). How plants respond to elevated CO_2_ and its role as a key substrate for photosynthesis are closely connected to these processes (Blatt, 2000; Lawson, 2009; Zhang et al., 2018a; Papanatsiou et al., 2019). Land plants take up CO_2_ by diffusion through stomatal pores that are present in the leaf epidermis. CO_2_ uptake necessarily occurs in exchange with water vapor, which is lost via transpiration from the inner leaf airspace through the stomatal pore to the atmosphere (Lawson and Blatt, 2014). Stomatal aperture is thus a focal point both for photosynthesis and for water balance within the leaf tissues (Blatt, 2000; Meckel et al., 2007; Lawson and Blatt, 2014; Jezek and Blatt, 2017).

Stomata open and close through dynamic changes in volume and turgor of the surrounding guard cells driven by solute and water transport across the guard cell plasma membrane and tonoplast (Lawson and Blatt, 2014; Jezek and Blatt, 2017). H^+^‐ATPases generate gradients in pH and membrane voltage at both membranes that energize the transport of osmotically active solutes—mainly of potassium (K^+^), chloride (Cl^−^), and malate (Mal) ions—to draw water into the cell, generating turgor and affecting the guard cell volume (Blatt, 2000, 2024; Meckel et al., 2007; Sutter et al., 2007; Lawson and Blatt, 2014; Brodribb and McAdam, 2017; Jezek and Blatt, 2017). Among the transporters at the plasma membrane are the inward‐rectifying K^+^ channels, POTASSIUM CHANNEL IN ARABIDOPSIS THALIANA (KAT1, KAT2), which mediates K^+^ uptake for stomatal opening (Blatt, 2000; Jezek and Blatt, 2017; Zhang et al., 2018a), the guard cell outward‐rectifying K^+^ channel (GORK) and S‐type or SLOW ANION CHANNEL‐ASSOCIATED (SLAC)‐type (SLAC1, SLAH3) anion channels that facilitate K^+^ and Cl^−^ efflux for stomatal closure (Ache et al., 2000; Hosy et al., 2003; Zhang et al., 2025). The activities of these ion channels and the plasma membrane H^+^‐ATPases are highly regulated, underpinning stomatal responses to light, the water‐stress hormone abscisic acid (ABA), and CO_2_, among others (Negi et al., 2008; Zhang et al., 2016; Jezek and Blatt, 2017; Zhang et al., 2018a; Blatt et al., 2022; Blatt, 2024).

While much interest is centered on the activities of transporters present at the membrane, osmotic solute transport is also subject to changes in transporter density through the activity of membrane vesicle traffic, and exo‐ and endocytosis (Eisenach et al., 2012; Jezek and Blatt, 2017; Karnik et al., 2017; Xia et al., 2019). Available evidence shows that vesicle traffic selectively adds and removes transport proteins, thereby affecting the overall capacity for solute flux (Eisenach et al., 2012; Lawson and Blatt, 2014; Jezek and Blatt, 2017; Xue et al., 2018; Xia et al., 2019; Baena et al., 2022, 2024; Rajappa et al., 2024). Vesicle trafficking in plant cells, as in all eukaryotes, is mediated by several superfamilies of eukaryotic proteins, notably the so‐called SNARE proteins that drive the final stages of fusion between the vesicle and target membrane bilayers (Sollner and Rothman, 1996; Jahn and Sudhof, 1999; Jürgens, 2004; Bassham and Blatt, 2008). SNARE action affects key transport proteins, including the plasma membrane H^+^‐ATPases (Jezek and Blatt, 2017; Xia et al., 2019; Fuglsang and Palmgren, 2021; Baena et al., 2022), K^+^ channels (Sutter et al., 2006; Sutter et al., 2007; Eisenach et al., 2012; Lefoulon et al., 2018), and aquaporins (Hachez et al., 2013, 2014; Hachez et al., 2014). KAT1 K^+^ channel trafficking, for example, responds to the water‐stress hormone abscisic acid (ABA), affecting the subsequent re‐opening of stomata, and gives rise to the memory‐like behavior of so‐called “programmed closure” (Sutter et al., 2006; Eisenach et al., 2012).

As a major environmental factor to affect stomatal movements, we also know much about how CO_2_ affects transport in guard cells (Lawson and Blatt, 2014; Zhang et al., 2018a; Blatt et al., 2022). Aspects of transport regulation by CO_2_ overlap with those of the well‐studied response to abscisic acid (ABA) that, like elevated CO_2_, promotes stomatal closure (Assmann and Jegla, 2016; Jezek and Blatt, 2017). Among others, KAT1 channel activities in elevated CO_2_ and in ABA overlap, although there are differences in the subsets of protein kinases contributing to the channel regulation (Schulze et al., 2021). To date, however, our knowledge of CO_2_ action on vesicle traffic, especially its impact on ion transporters, is sparse. Indeed, there is no information on whether traffic is affected by elevated CO_2_ and, if so, how it may impact on stomatal function and, more broadly, on vegetative physiology.

We have examined the traffic of the KAT1 K^+^ channel that was previously described to cycle from the plasma membrane to vesicular compartments in response to both ABA and cytosolic‐free [Ca^2+^] ([Ca^2+^]_i_) elevations (Sutter et al., 2007; Eisenach et al., 2012). The results uncover a dynamic in KAT1 mobility within the plasma membrane as well as effects on its distribution between the plasma membrane and endomembrane fractions. The effects of elevated CO_2_ are evident within minutes and are subject to the plasma membrane SNARE SYP121. While there are parallels to traffic of KAT1 in ABA, there are also clear differences in trafficking responses between the two closing stimuli. These, and additional data, demonstrate that SNARE‐dependent membrane traffic responds to elevated CO_2_, but its influence on plant growth and stomatal behavior is separable from that of ABA.

## RESULTS

### KAT1 punctal distribution is sensitive to CO_2_/HCO_3_^−^

As a vesicle trafficking cargo, we used the K^+^ channel KAT1, which is known to be affected by ABA and contributes to K^+^ uptake for stomatal opening (Sutter et al., 2006; Sutter et al., 2007; Zhong et al., 2024). *KAT1* is expressed widely in the plant, and it is highly abundant in Arabidopsis leaf tissue, particularly the epidermis and guard cells (Nakamura et al., 1995; Figure S1A; Table S1). KAT1 forms small clusters that are normally immobile and distributed over the plasma membrane surface, visible as fluorescent puncta with the xFP‐tagged channel (Sutter et al., 2006).

**Figure 1:**
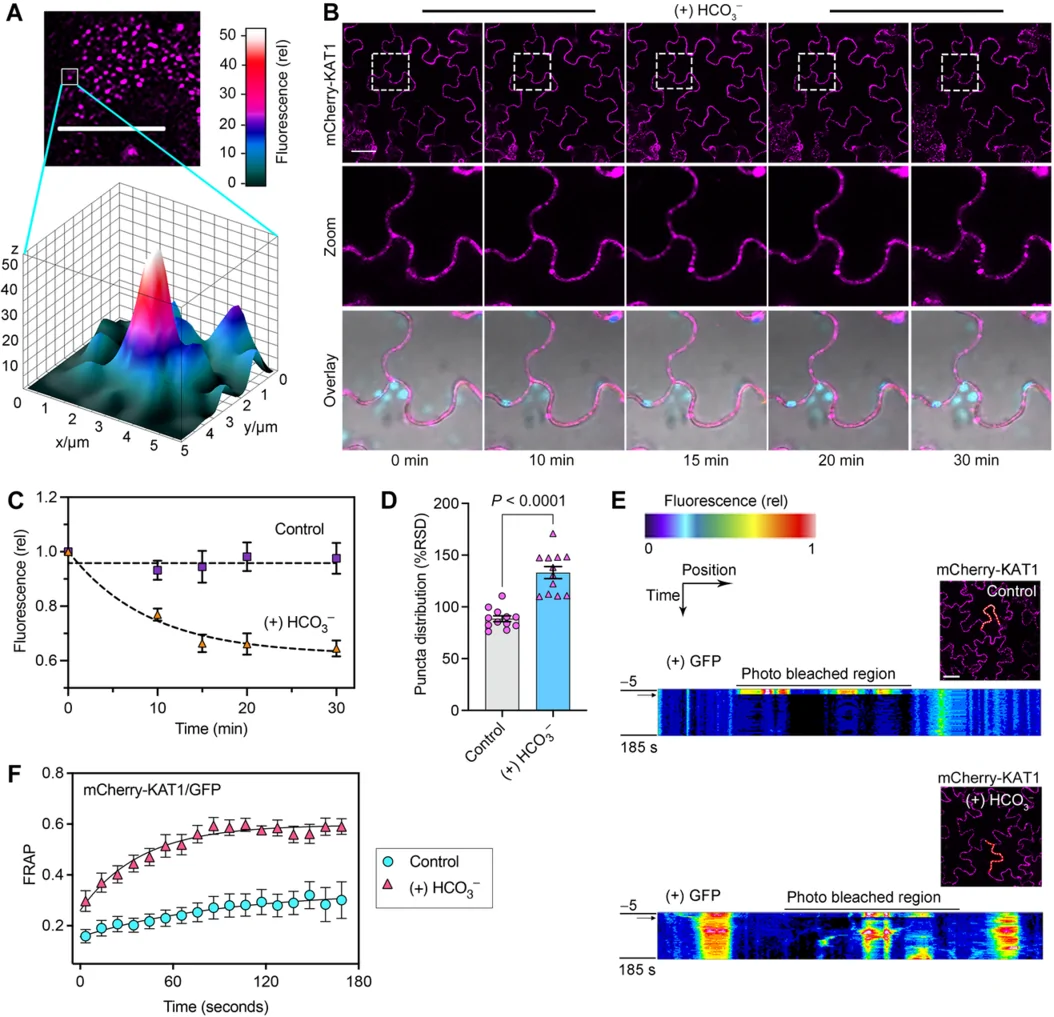
KAT1 punctal distribution is sensitive to CO_2_/HCO_3_^−^ **(A)** Three‐dimensional (3D) surface plot of a single mCherry‐KAT1 cluster from a confocal microscope image of *Nicotiana tobaccum* epidermis, where the x‐ and y‐axes show the image coordinates, and the z‐axis shows the fluorescence intensity. Fluorescence is represented using pseudo‐color (see the scale) and was well fitted to a two‐dimensional Gaussian distribution (Figure S1). **(B)** Representative confocal images of *Nicotiana tabacum* epidermal cells expressing mCherry‐KAT1 acquired on a single focal plane at 0‐, 10‐, 15‐, 20‐, and 30‐min intervals following treatment with water (control) or 1 mM HCO_3_^−^ [(+) HCO_3_^−^]. Images show mCherry‐KAT1 fluorescence (top panels) with region of interest (ROI) (white dashed line), zoomed ROI images (middle panels), and overlay showing bright‐field and chlorophyll fluorescence (bottom panels). Scale bar = 50 μm (also see Figure S2). **(C)** Means ± *SE* mCherry‐KAT1 fluorescence analysis along cell periphery (*n* ≥ 12) images, see **(B)**, relative to the control (0 min), see Methods. Fluorescence decay, halftime (*t*_1/2_) = 9.2 ± 1.1 min, following 1 mM bicarbonate treatment, calculated using nonlinear least‐squares fit as single exponential decay. **(D)** Mean %Relative standard deviation (RSD) ± *SE* of mCherry‐KAT1 fluorescence distribution along the cell periphery at 30 min in control and (+) HCO_3_^−^‐treated cells **(B)**; see Methods. Statistical significance was determined using the Mann–Whitney test for cumulative distributions from *n* = 12 data sets. *P* values are indicated. **(E)** Kymographic analysis of mCherry‐KAT1 fluorescence intensity (pseudo‐colored; see the scale) in microdomains (2 × 150 μm ROI) along the cell periphery (see the representative image and inset, dotted line) plotted against time (seconds) on the x‐axis. The arrow on the left indicates the bleaching time. Scale bar = 50 μm. **(F)** Means ± *SE* mCherry‐KAT1 fluorescence signal recovery post‐bleach in control (squares) and (+) HCO_3_^−^ (triangles)‐treated cells depicted as relative to pre‐bleach fluorescence; see **(E)**. Data from ≥ 6 datasets are shown, fitted by nonlinear least squares to a single‐exponential function (bold lines). Fluorescence recovery half‐times (*t*_1/2_) were 82 ± 23 s and 18 ± 2 s, respectively. Statistical significance was determined using the Mann–Whitney test (*P* < 0.001), *n* = 3.

When expressed in *Nicotiana tobaccum* leaf epidermis, we observed mCherry‐tagged KAT1 similarly to form fluorescent puncta at the cell periphery (Figure 1A). Analysis of these puncta indicated discrete and near isodiametric fluorescence distributions, with a full width at half‐maximum intensity (FWHM) of 1.19 ± 0.25 μm (Figure S1B), much as reported previously for the channel (Sutter et al., 2006). The mCherry label was not observed to accumulate in the endoplasmic reticulum (Figure 1A, B), suggesting that the channel distribution was largely unaffected despite overexpression. As in past studies (Sutter et al., 2006; Sutter et al., 2007), we therefore used tobacco epidermis to track KAT1 traffic with CO_2_.

Gaseous CO_2_ forms carbonic acid with water, the acid then dissociating to form HCO_3_^−^ and releasing H^+^ at near‐neutral pH. The HCO_3_^−^ anion is thought to be an active ligand controlling stomatal movements within the plant (Lawson, 2009; Zhang et al., 2018b). At atmospheric partial pressure and 20°C temperature, CO_2_ dissolves to yield 12 μM CO_2_ in solution and equilibrates to yield 0.22 mM HCO_3_^−^ at pH 7.5. Thus, 1 mM HCO_3_^−^ in solution at pH 7.5 equates to an atmospheric CO_2_ partial pressure some four‐ to fivefold above ambient CO_2_.

We measured mCherry‐KAT1 at the cell periphery using the bright‐field image overlay for each experiment to establish the cell perimeter. Images were collected at the start of treatments (*t* = 0; see Methods) and at time points of 10, 15, 20, and 30 min both with water as a control and with 1 mM bicarbonate [(+) HCO_3_^−^] to evaluate the effects of elevated CO_2_ signals on the channels (Figures 1B, S2A). The mCherry‐KAT1 fluorescence was stable in the control but decreased with a half‐time (*t*_1/2_) of 9.2 ± 1.1 min in 1 mM HCO_3_^−^ (Figures 1C, S2A). The changes in mCherry‐KAT1 fluorescence were sensitive to the [HCO_3_^−^] concentration and suggested a decrease in channel density at the cell periphery in response to elevated CO_2_ (Figure S2B, C). Decreases in mCherry‐KAT1 fluorescence were observed in both 1 mM and 3 mM HCO_3_^−^‐treated cells after 15 min. By contrast, additions of 0.1 mM HCO_3_^−^ equivalent to sub‐ambient atmospheric CO_2_ partial pressure near 200 μbar led to an increase in mCherry‐KAT1 fluorescence (Figure S2B, C). Fitting the complement of the steady‐state fluorescence with [HCO_3_^−^] to a hyperbolic function yielded a dependence with an apparent *K*_1/2_ of 0.48 ± 0.03, consistent with the range of CO_2_/HCO_3_^−^ over which stomata normally respond (Cowan and Farquhar, 1977; Mott, 1990). As an additional control, we challenged leaf tissues with 20 μM ABA (Figure S3) and followed mCherry‐KAT1 fluorescence over time. In this case, the fluorescence signal decayed with a half‐time of 8.7 ±1.4 min, a rate kinetically indistinguishable from that of the response to 1 mM HCO_3_^−^ (Figure S3A, B).

ABA treatment was shown previously to promote KAT1 mobility in the membrane and endocytosis from focal points represented by the puncta (Sutter et al., 2007). We asked, therefore, whether HCO_3_^−^ similarly affected the punctal fluorescence distribution of KAT1. To this end, we analyzed confocal images collected over time in HCO_3_^−^ and quantified the mean relative standard deviation (RSD) along the cell periphery (Figure 1D). Changes in RSD will arise when the fluorophore‐tagged protein is redistributed, the RSD decreasing as the punctal fluorescence declines and spreads at the cell surface (Eisenach et al., 2014; Xia et al., 2019). Surprisingly, we found that the mCherry‐KAT1 RSD increased significantly following exposures to 1 mM HCO_3_^−^ compared to the control (Figures 1D, S4). For comparison, we repeated these experiments, adding 20 μM ABA to monitor mCherry‐KAT1 fluorescence decay and quantify the associated RSD. The results (Figure S3C) showed that ABA, by contrast, led to a decline in punctal fluorescence with a reduction in RSD. Thus, although the overall fluorescence at the periphery decreased in HCO_3_^−^ (Figures 1D, S4C), the net effect was to enhance the apparent punctate distribution of KAT1, unlike the effects of ABA (Sutter et al., 2007), as if HCO_3_^−^ promoted the removal of channels in a manner that is not constrained to the focal points of the puncta.

As a further test of mCherry‐KAT1 mobility in HCO_3_^−^, we examined the fluorescence recovery after photobleaching (FRAP) of the mCherry fluorophore locally and followed its recovery over time. As photobleaching is not reversible, fluorescence recovery must arise from relocation of non‐bleached mCherry‐KAT1 into the zone previously bleached during the experiment. As expected, kymographic analysis (Figure 1E; see the inset) along the cell periphery from image time series showed little evidence of mobility after bleaching in the control. However, the mCherry‐KAT1 fluorescence recovered rapidly when tissues were exposed to 1 mM HCO_3_^−^ (Figure 1F). Half‐times for recovery derived from control and HCO_3_^−^ treatments were 82 ± 23 s and 18 ± 2 s, respectively (*n* ≥5). In both cases, distal fluorescence signals away from the photobleached regions were not appreciably affected (Figure S5), indicating that the fluorescence kinetics arose with fluorophore relocation, not from a general decay in signal.

Finally, we prepared membrane fractions from leaves expressing mCherry‐KAT1 infiltrated with water and with 1 mM HCO_3_^−^ for 30 min prior to isolation. Plasma membrane (PM) and internal membrane (IM)‐enriched fractions were separated using a membrane fractionation kit that includes differential centrifugation for membrane fractionation (see Methods). The quality of the membrane fractionation was assessed using antibodies to the endoplasmic reticulum resident lumen‐binding protein (BiP) as the reference. BiP was detected in the internal membrane fraction (IM) but was absent in the plasma membrane (PM) fraction, indicating that the membrane preparations were of high purity (> 99%). Microsomal total, PM, and IM samples for each treatment were prepared by solubilizing equal amounts of total membrane protein in Laemmli buffer and resolved by SDS‐PAGE prior to immunoblotting (Figure S6A). mCherry‐KAT1 protein was detected using anti‐mCherry antibodies, and the band intensity in water‐ and HCO_3_^−^‐treated samples was calculated relative to the total protein in each lane (see Methods, Baena et al., 2022). Following HCO_3_^−^ treatment, a twofold reduction in channel abundance in the plasma membrane fraction relative to the control was observed, while channel density in the internal membrane fractions was significantly higher (Figure S6A, B). Overall, the results indicated that elevated HCO_3_^−^ in solution promotes KAT1 mobility, including at the punctal foci at the plasma membrane, such that channel endocytosis suppresses its abundance at the plasma membrane.

### SYP121 affects KAT1 distribution with CO_2_

The Arabidopsis SNARE protein SYP121 (SYR1/PEN1) is one of two predominant SNARE proteins driving vesicle traffic at the plasma membrane (Karnik et al., 2015). SYP121 represents important stomatal responsiveness and KAT1 cycling in ABA (Leyman et al., 1999; Sutter et al., 2006; Sutter et al., 2007) and it underpins the kinetics and so‐called “programmed closure” of stomata (Eisenach et al., 2012). SYP121 also interacts physically with KAT1 at the plasma membrane to promote the channel activity as well as SYP121‐mediated secretory vesicle traffic (Grefen et al., 2010a, 2015). Thus, we asked whether SYP121 might also play a role in KAT1 redistributions in response to HCO_3_^−^.

**Figure 2:**
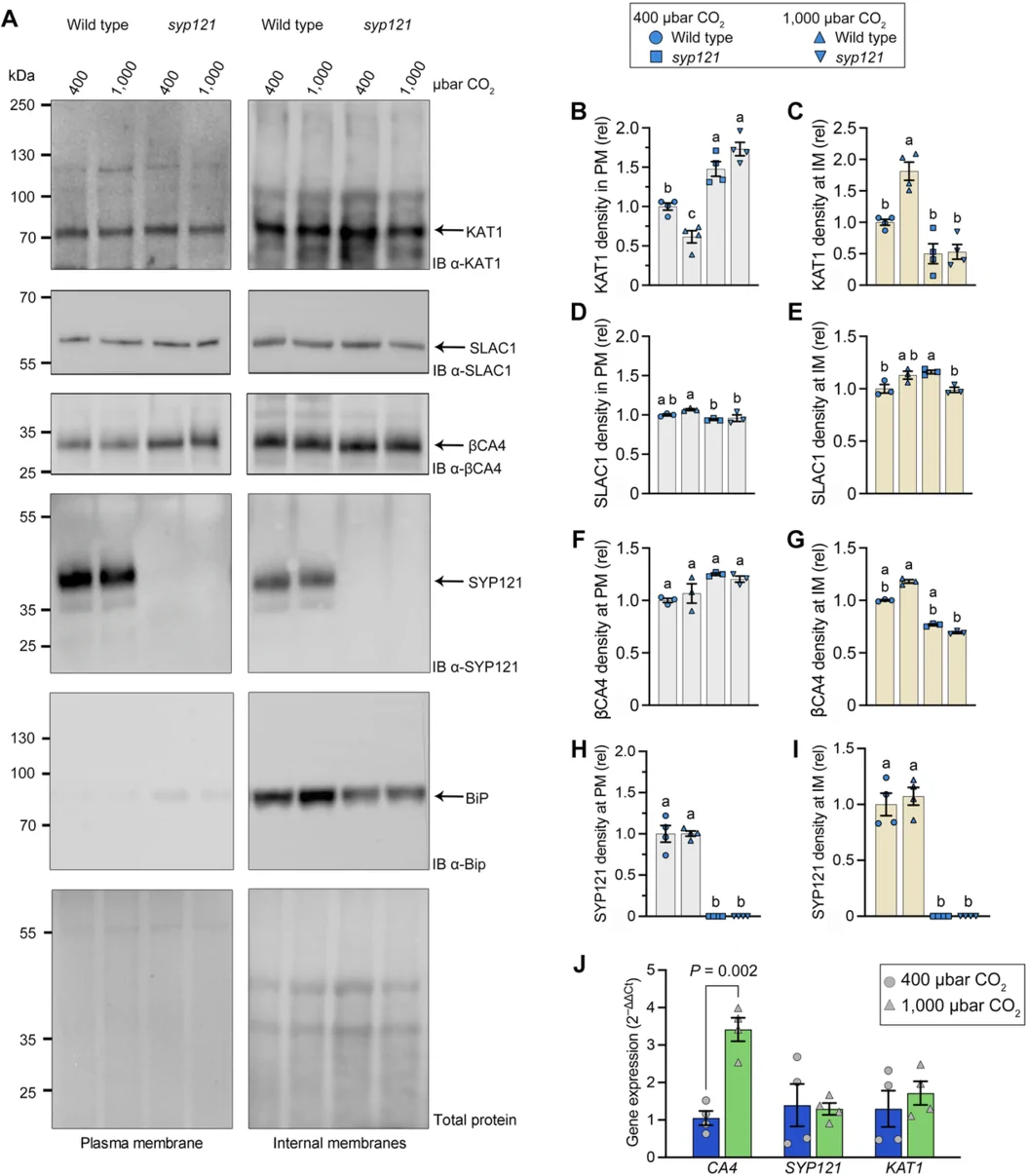
CO_2_ regulates KAT1 distribution at the plasma membrane, independent of gene transcription **(A)** Immunoblots of plasma membrane and inner membrane fractions purified from rosette leaves of wild‐type and *syp121* Arabidopsis in response to ambient (400 μbar) or elevated (1,000 μbar) CO_2_. Proteins were resolved on a single gel (10% polyacrylamide SDS‐PAGE) and detected by immunoblotting with antibodies against specific native proteins, anti‐KAT1 (KAT1 K^+^ channel, 78 kDa; first panel), anti‐SLAC1 (SLAC1 Cl^−^ channel, 63.2 kDa; second panel), anti‐βCA4 (βcarbonic anhydrase 4, 30.8 kDa; third panel), anti‐SYP121 (SYP121, 38 kDa; fourth panel), and anti‐BiP (lumen‐binding protein, 73.5 kDa; fifth panel). Also see Figures S6, S7. Black lines (left) indicate the positions of molecular mass markers and black arrows (right) indicate the expected band positions. Total protein in each lane was detected using Coomassie blue stain (bottom panel) and used for quantitative analysis (see Materials and Methods). Based on band intensities for BiP, 97.1% ± 0.4% of the marker appeared in the inner membrane fractions (*n* ≥ 3). **(B**–**I)** Relative KAT1, SLAC1, βCA4, and SYP121 distribution in the plasma membrane and inner membrane of wild‐type and *syp121* Arabidopsis leaves quantified from immunoblots using ImageJ software. Data are means ± *SE*, normalizing with wild‐type membrane fractionations under 400 μbar CO_2_. Statistical significance was assessed using the one‐way ANOVA method with Tukey's multiple comparisons test (*P* < 0.05), *n* ≥ 3. **(J)** RT‐qPCR analysis is shown with mitochondrial 18s rRNA (AtMg01390) expression as the internal reference on leaves from 3–4‐week‐old Arabidopsis wild type using gene‐specific primers (Table S1). Relative βCA4, SYP121, and KAT1 expressions (2^−ΔΔCt^) for control (circles, 0 h, 400 μbar CO_2_) and treated samples (triangles, 24 h, 1,000 μbar CO_2_) are shown. Data are means ± *SE*. Statistically significant differences are indicated with a *P* value using the Holm–Šídák multiple comparison test (*P* < 0.05), *n* = 4.

We analyzed leaf membrane fractions from wild‐type and *syp121* null mutant Arabidopsis subjected to 400 and 1,000 μbar atmospheric CO_2_ partial pressure for 30 min prior to extraction. These concentrations represent ambient and elevated CO_2_ environments, respectively. In Arabidopsis leaves, KAT1 is predominantly expressed in the epidermal and guard cells (Figure S1A; Nakamura et al., 1995). Plasma membrane and internal membrane fractions were prepared as above (Figure S6) and analyzed using custom antibodies (Figure S7) detecting native Arabidopsis KAT1 (Figure 2). We found that KAT1 abundance at the plasma membrane declined by approximately twofold in wild‐type plants when challenged with 1,000 μbar CO_2_ (Figure 2A, B), with a corresponding increase within the IM fraction (Figure 2A, C). Overall, the KAT1 density was significantly higher in *syp121* null mutants, with the majority of proteins localized to the plasma membrane in both ambient and elevated CO_2_ (Figure 2A, B).

We conducted immunoblot analysis to track native SLAC1, a predominant guard cell anion channel in stomatal closure (Vahisalu et al., 2008). These experiments showed no obvious CO_2_‐dependent changes in protein abundance at the plasma membrane in both wild‐type and *syp121* mutant plants (Figure 2A, D, E). We also tested if there was any change in the density of βcarbonic anhydrase 4 (βCA4) protein that catalyzes the reversible hydration of CO_2_ to carbonic acid and influences stomatal responsiveness to CO_2_ (Fabre et al., 2007; Hu et al., 2010; DiMario et al., 2017). Immunoblots using custom antibodies against βCA4 (Figure S7B, C) indicated modest changes in protein abundance but only in *syp121* mutant plants, with the internal membrane fraction showing an approximately 25% decrease in βCA4 density in elevated CO_2_ (Figure 2A, F, G). In parallel, we tested for changes in SYP121 density with custom antibodies (Tyrrell et al., 2007). The results showed that elevated CO_2_ had no significant effect on SYP121 abundance in either plasma or internal membranes (Figure 2A, H, I). Thus, in elevated CO_2_ conditions, the changes in protein abundance at the plasma membrane appeared to be specific to KAT1.

Finally, we tested whether the changes in KAT1 density at the plasma membrane might arise with gene expression. We carried out qRT‐PCR analysis of leaves using gene‐specific primers for *SYP121*, *KAT1*, and CA4 after exposing plants to 400 μbar and 1,000 μbar CO_2_ for 24 h. The results showed that neither *KAT1* nor *SYP121* was affected by elevated CO_2_; only *βCA4* expression increased appreciably with elevated CO_2_ (Figure 2J; Table S1). We also observed little effect of the *syp121* null mutant on *KAT1* expression compared to its close homolog *KAT2* and the K^+^ co‐transporter *HAK5*; only in the *kat1* null mutant did we note a significant increase in *KAT2* and *HAK5* expression (Figure S8). Altogether, these data suggest that the actions of CO_2_ are selective for the KAT1 protein and are not substantially affected by changes in transcript abundance.

### SYP121 affects KAT1 mobility with CO_2_ *in vivo*

**Figure 3:**
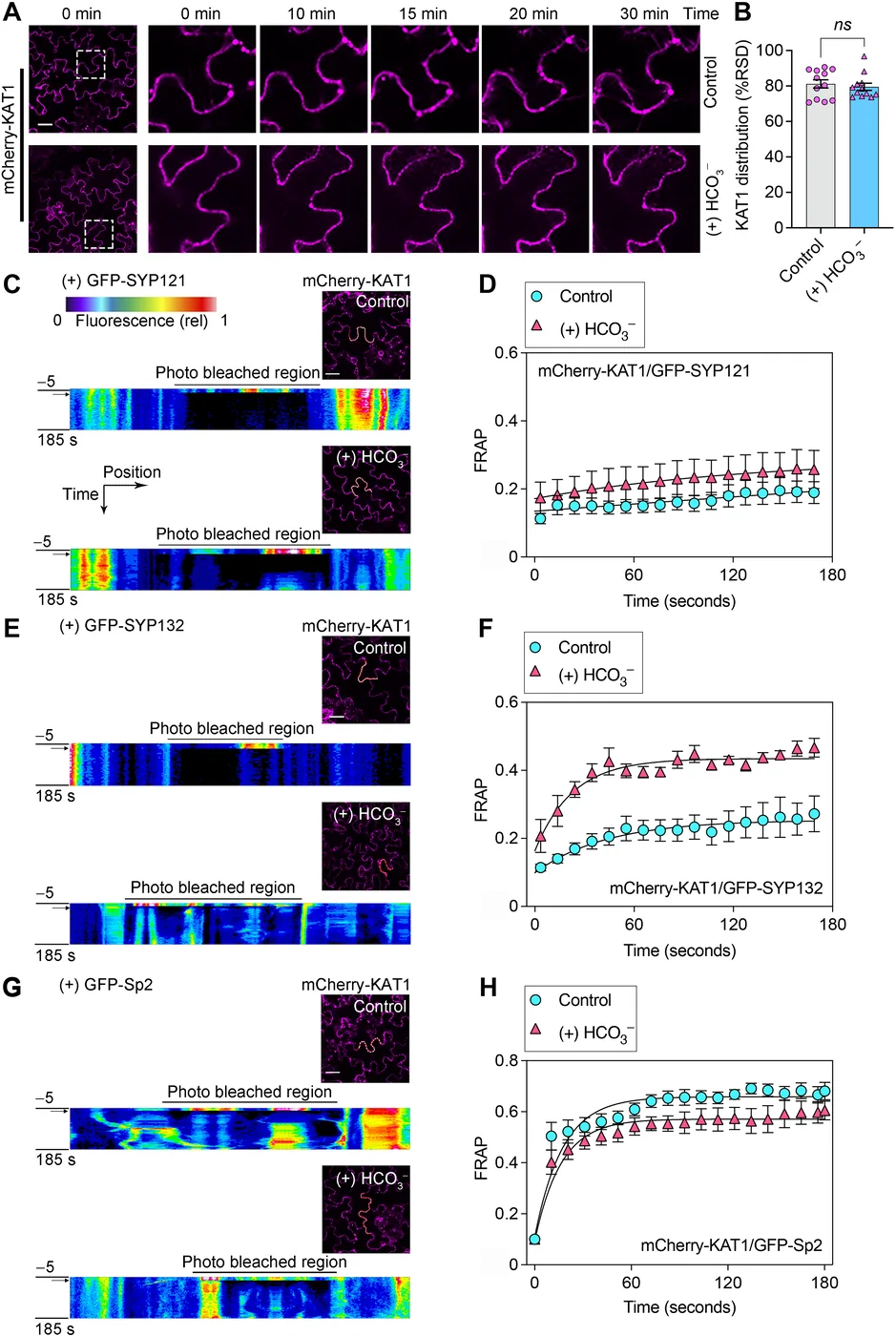
SYP121 affects KAT1 mobility with CO_2_ *in vivo* **(A)** Confocal images, representative of *Nicotiana tabacum* epidermal cells co‐expressing mCherry‐KAT1 and GFP‐SYP121, acquired on a single focal plane at 0‐, 10‐, 15‐, 20‐, and 30‐min intervals, following treatment with water (control) or 1 mM HCO_3_^−^ [(+) HCO_3_^−^] extracted from wider field images (Figure S9). mCherry‐KAT1 fluorescence within the region of interest (ROI) (white dashed line) is marked (wide‐field, 0 min) and zoomed ROI images are shown. Scale bar = 50 μm. **(B)** Mean relative standard deviation (%RSD) ± *SE* mCherry‐KAT1 fluorescence distribution in cells co‐expressing GFP‐SYP121 after 30 min of control or (+) HCO_3_^−^ treatments, see **(A)**, measured across a 1.5‐pixel‐wide line along the periphery of cells (see methods) from > 5 image sets each (*n* = 12). Statistical analysis compares cumulative distributions using the Mann–Whitney test (*P* < 0.05). **(C**–**H)** Kymographic analysis (left panels) of mCherry‐KAT1 fluorescence intensity (pseudo‐colored;  see the scale) plotted against time (seconds). Measurements were from microdomains (2 × 150 μm ROI; see the inset yellow dotted line) along the periphery of epidermal cells co‐expressing GFP‐SYP121 **(C)**, GFP‐SYP132 **(E)**, or GFP‐Sp2 fragment **(G)**. Representative confocal images are shown. The arrow on the left indicates the bleaching time. Scale bar = 50 μm. Means ± *SE* mCherry‐KAT1 fluorescence signal recovery post‐bleach (right panels) in control (water, circles) and (+) HCO_3_^−^ (triangles)‐treated cells co‐expressing GFP‐SYP121 **(D)**, GFP‐SYP132 **(F)**, or GFP‐Sp2 fragment **(H)** relative to pre‐bleach fluorescence. Fluorescence recovery half‐times (*t*_1/2_) were calculated for GFP‐SYP132 as 45 ± 11 s (control) and 19 ± 5.6 s (1 mM HCO_3_^−^) and for the GFP‐Sp2 fragment as 13.4 ± 2.8 s (control) and 12 ± 3.1 s (1 mM HCO_3_^−^), respectively, using ≥ 5 data sets. Data are fitted by nonlinear least squares to a single‐exponential function (bold lines), *n* = 3.

To assess the effects of SYP121 *in vivo*, we co‐expressed mCherry‐KAT1 and GFP‐tagged SYP121 in tobacco leaf epidermis. As KAT1 recycling is slowed in *syp121* mutant Arabidopsis (Eisenach et al., 2012), we anticipated that expressing the SNARE might enhance KAT1 traffic. Unexpectedly, we found that co‐expressing SYP121 appears to stabilize the K^+^ channels and inhibit channel redistribution in response to elevated HCO_3_^−^. We observed no significant changes in total mCherry‐KAT1 fluorescence (Figures 3A, S9) or in fluorescence RSD along the cell periphery when comparing control tissues and those treated with 1 mM HCO_3_^−^ (Figure 3B). Accompanying immunoblot assays showed that relative to the control, 1 mM HCO_3_^−^ treatment had no effect on KAT1 or SYP121 protein abundance (Figure S10). As a test for KAT1 mobility with SYP121, we also used FRAP analysis as described above. These experiments (Figure 3C, D) showed that in cells expressing GFP‐SYP121, the mCherry‐KAT1 fluorescence signal did not recover after local photobleaching (*t*_1/2_ > 180 s) either in the control or in tissues treated with 1 mM HCO_3_^−^.

To test the specificity of SYP121 action in HCO_3_^−^, the same measurements were carried out using the close SYP121 homolog, SYP132, one of three SNAREs active at the plasma membrane (Waghmare et al., 2024). SYP132 is normally present at much lower levels than SYP121 but is essential for traffic of the dominant H^+^‐ATPase and aquaporin PiP2;1 (Xia et al., 2019; Baena et al., 2024) and has no influence on KAT1 traffic (Xia et al., 2019). After co‐expressing mCherry‐KAT1 with GFP‐SYP132, we found (Figure 3E, F) that mCherry‐KAT1 fluorescence recovered was significantly faster in FRAP experiments with 1 mM HCO_3_^−^ (*t*_1/2_ = 19 ± 5.6 s) than in the control (*t*_1/2_ = 45 ± 11 s), much as we observed in the absence of SYP121 and consistent with the idea that SYP121 action is specific for KAT1.

We also tested the effects of the truncated form of SYP121, SYP121^ΔC^ (SYP121‐Sp2), which lacks the C‐terminal membrane anchor and is known to act as a dominant‐negative competitor of SYP121 *in vivo* (Geelen et al., 2002; Tyrrell et al., 2007). Co‐expression of SYP121^ΔC^ is known to affect KAT1 recycling as well as its positional stability within the plasma membrane (Sutter et al., 2006). On co‐expression with mCherry‐KAT1, we found that SYP121^ΔC^ enhanced the recovery in KAT1 fluorescence following photobleaching both in the control (*t*_1/2_ = 13.38 ± 2.8 s) and in tissues treated with 1 mM HCO_3_^−^ (*t*_1/2_ = 11.95 ± 3.1 s) (Figures 3G, H, S11). Overall, these findings suggest that KAT1 anchoring and turnover with CO_2_/HCO_3_^−^ are subject to traffic mediated by SYP121 at the plasma membrane.

### KAT1 clustering with CO_2_/HCO_3_^−^ is SNARE‐dependent

Previous studies showed that KAT1 puncta are centers for exo‐ and endocytic traffic that regulate the active pool of channels at the plasma membrane (Sutter et al., 2006; Sutter et al., 2007). The K^+^ channel protein localized within puncta are positionally stable and, at least in ABA, the tagged channel is internalized without redistribution over the cell surface (Sutter et al., 2007; Xia et al., 2019). We expected elevated CO_2_/HCO_3_^−^ to promote KAT1 endocytosis and suppress channel density at the cell periphery and were thus surprised to find that the puncta became more pronounced, as indicated by the increase in RSD (Figure 1).

**Figure 4:**
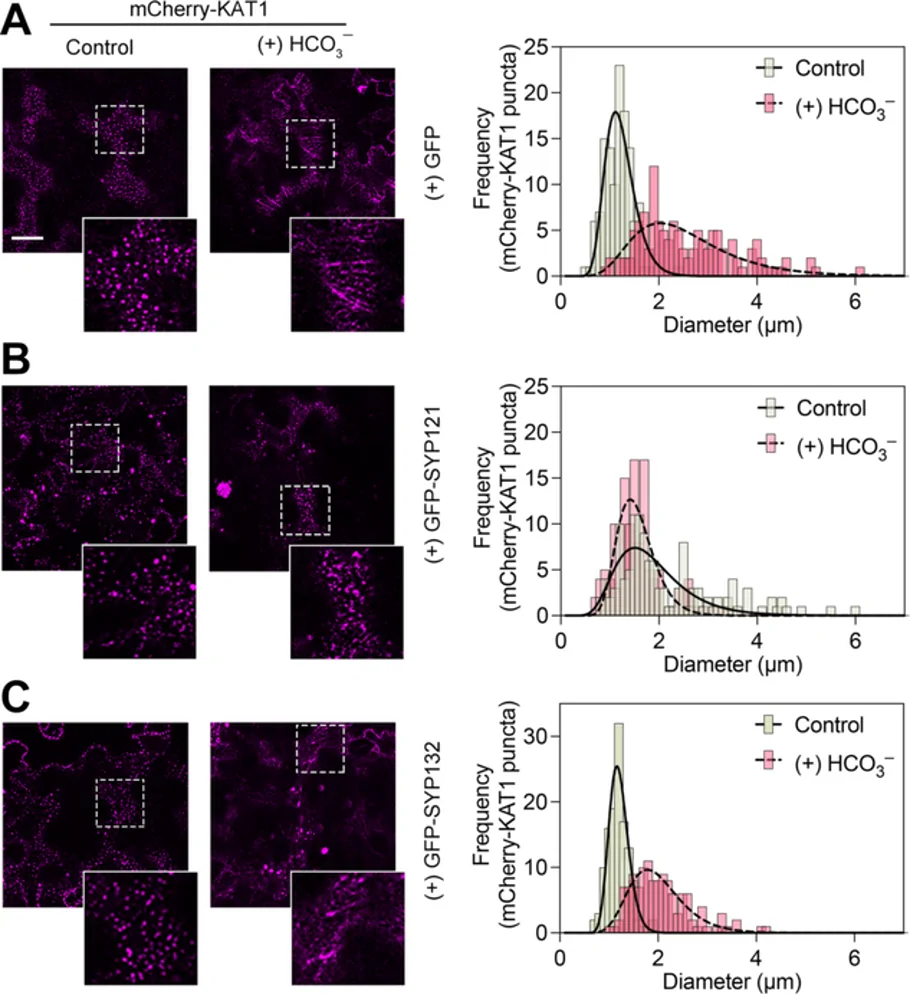
SYP121 affects KAT1 punctation **(A**–**C)** Confocal images (representative) acquired along a single plane at the cell surface in *Nicotiana tabacum* epidermis co‐expressing mCherry‐KAT1 with GFP **(A)**, GFP‐SYP121 **(B)**, or GFP‐SYP132 **(C)**. Images show mCherry‐KAT1 fluorescence (left panels) following control or 1 mM HCO_3_^−^ treatment (30 min), with the region of interest (ROI) marked (white dashed line) and zoomed ROI images showing wide‐field figures (see Figure S12A, bright‐field overlay). Scale bar = 50 μm. Frequency histograms of mCherry‐KAT1 puncta diameters (right panels) for control (solid curve) and (+) HCO_3_^−^ (dotted curve) treatments are shown. Data are 200 puncta diameters from ≥ 6 images (*n* = 3) fitted to Gaussian functions using a Levenberg–Marquardt algorithm (Marquardt, 1963) for nonlinear least squares to extract peak and FWHM dimensions. Peak geometric mean distributions ± *SE* for control and HCO_3_^−^ treatments, respectively, were 1.2 ± 0.24 μm and 2.4 ± 0.40 μm (KAT1), 1.74 ± 0.37 μm and 1.5 ± 0.24 μm (KAT1 + GFP‐SYP121), or 1.2 ± 0.16 μm and 1.9 ± 0.28 μm (KAT1 + GFP‐SYP132). Transient protein expressions were verified by immunoblotting (Figure S12).

To explore this question in greater depth, we measured the FWHM diameters of randomly selected puncta and analyzed the data for puncta size distributions both with mCherry‐KAT1 co‐expressed with GFP as a control and with the GFP‐tagged SNAREs described above. Again, we used transiently transformed tobacco epidermis and verified protein expression by immunoblot (Figure S12). Frequency histograms showed that mCherry‐KAT1 punctal size increased in HCO_3_^−^ (control, 1.19 ± 0.25 μm; HCO_3_^−^, 2.38 ± 0.40 μm)  (Figure 4A). In 1 mM HCO_3_^−^, the fluorescence expanded substantially, consistent with an increase in mobility within the membrane. By contrast, overexpression of GFP‐SYP121 (Figure 4B) yielded mCherry‐KAT1 puncta of marginally greater size that were not affected appreciably by HCO_3_^−^ (control, 1.74 ± 0.38 μm; HCO_3_^−^, 1.50 ± 0.25 μm). Finally, with GFP‐SYP132 co‐expression, we found no significant differences in either peak size or spread when compared to mCherry‐KAT1 expressed with GFP as a control (control, 1.19 ± 0.16 μm; HCO_3_^−^, 1.93 ± 0.28 μm) (Figure 4C). Thus, KAT1 puncta size and spread were affected by SYP121, much as we observed for channel mobility with elevated CO_2_/HCO_3_^−^.

**Figure 5:**
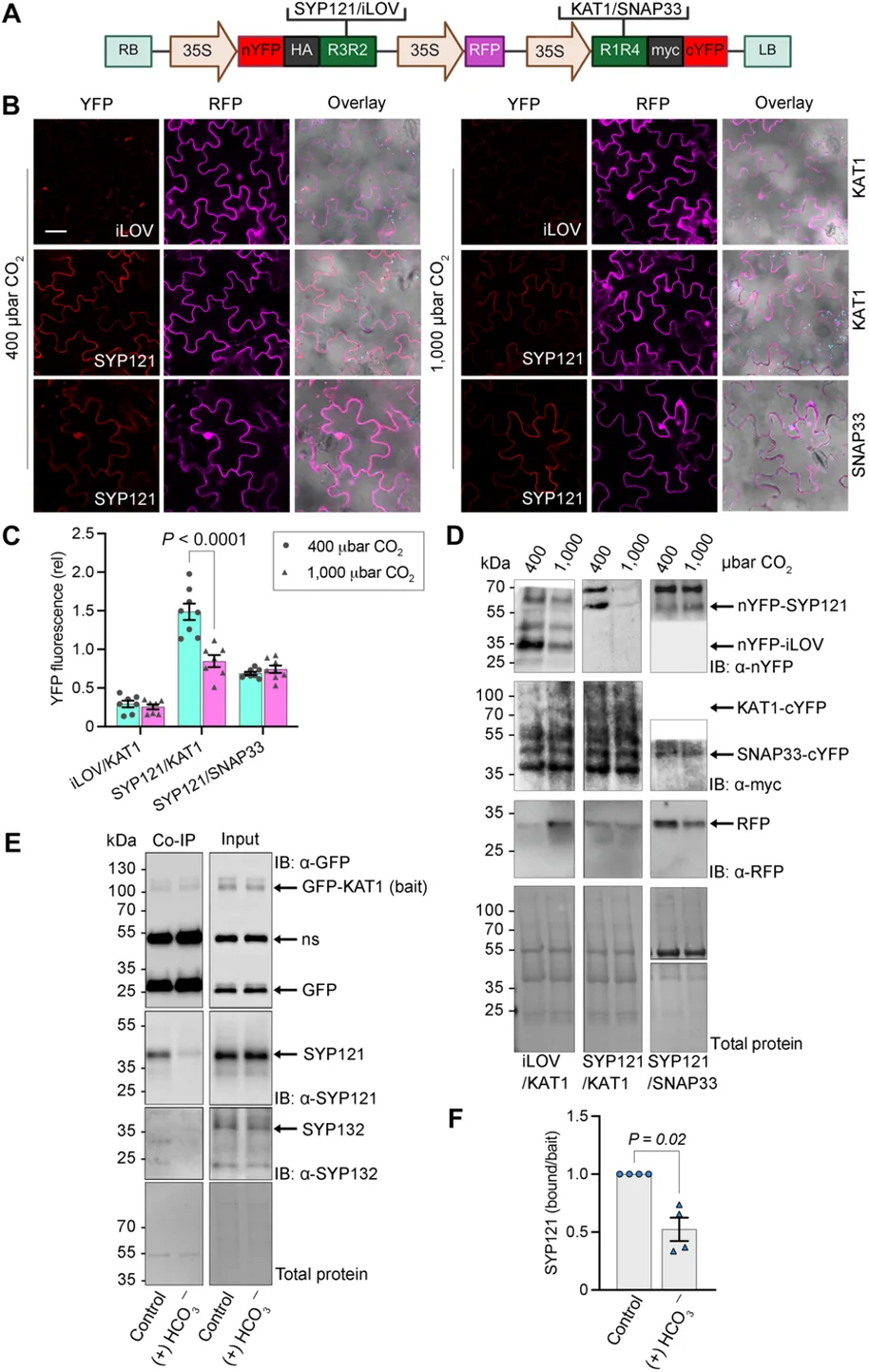
CO_2_ regulation of SYP121 binding with KAT1 **(A)** Schematic of the pBiFCt‐2in1‐NC and pBiFCt‐2in1‐CC vector containing SYP121or iLOV, and KAT1 or SNAP33. **(B)** Representative images of abaxial leaf epidermis cells of *Nicotiana tabacum* expressed pBiFCt‐2in1‐CC (iLOV/KAT1) (top panels), pBiFCt‐2in1‐NC (SYP121/KAT1) (middle panels), and pBiFCt‐2in1‐NC (SYP121/SNAP33) (bottom panels) under 400 μbar and 1,000 μbar CO_2_, respectively. Images show YFP fluorescence for the protein interaction (left), control images for RFP (middle), and an overlay of images with bright field, and chlorophyll (blue) fluorescence (right). Scale bar = 50 μm. **(C)** Quantification of the mean YFP fluorescence ratio against RFP resolves the selective interaction of KAT1 with SYP121, and iLOV or SNAP33 as a control. Data are means ± *SE* of ≥ 7 images of leaf epidermis (*n* = 3). Statistical significance for the data was determined using pairwise comparisons with Holm–Šídák's multiple comparisons test (*P* < 0.05). *P* values are indicated. **(D)** Immunoblots using anti‐myc and anti‐nYFP antibodies to verify protein expressions; nYFP‐SYP121 (58.7 kDa), nYFP‐iLOV (33.6 kDa), myc‐SNAP33 (47 kDa), and myc‐KAT1 (83.7 kDa). Total proteins were stained with Coomassie blue (bottom panels). Black lines (left) indicate the positions of molecular mass markers and black arrows (right) indicate expected band positions. **(E)** Representative immunoblots for co‐immunoprecipitation experiments with Arabidopsis transformed to express GFP‐KAT1 and incubated without and with 1 mM HCO_3_^−^. Blots are input and co‐immunoprecipitant GFP‐KAT1 (bait, 104 kDa) and bound SYP121 (38 kDa), SYP132 (35 kDa), detected using anti‐GFP, anti‐SYP121, and anti‐SYP132 antibodies, respectively. Coomassie blue stain was used to determine the total protein in each lane (bottom panels). GFP‐KAT1 bound SYP121 but not SYP132. **(F)** Graph means ± *SE* density of SYP121 bound/bait. Statistical significance was determined using a *t*‐test (*P* < 0.05). *P* values are indicated. *n* = 4.

KAT1, like the inward‐rectifying K^+^ channels KC1 and AKT1, interacts physically with SYP121 at the plasma membrane (Honsbein et al., 2009; Grefen et al., 2010a), and these interactions confer voltage sensitivity to vesicle trafficking in addition to regulating channel gating to promote K^+^ uptake (Grefen et al., 2015; Karnik et al., 2017). Given their importance, the question arose whether the SNARE‐channel interactions might be subject to CO_2_. We used ratiometric bimolecular fluorescence complementation (rBiFC) assays with the 2in1 multicistronic vector system (Grefen and Blatt, 2012; Karnik et al., 2013) to co‐express in tobacco epidermis C‐terminal YELLOW FLUORESCENT PROTEIN (cYFP)‐tagged KAT1 with N‐terminal YFP (nYFP)‐tagged SYP121 and, as negative and positive controls, with nYFP‐tagged iLOV (FLAVOPROTEIN IMPROVED LIGHT, OXYGEN, OR VOLTAGE DOMAIN PROTEIN) and SNAP33 (SOLUBLE N‐ETHYLMALEIMIDE‐SENSITIVE FACTOR ADAPTOR PROTEIN 33) (Karnik et al., 2013, 2015). The 2in1 system includes RED FLUORESCENT PROTEIN (RFP) as a marker for transformation and as a ratiometric reference for quantification (Figure 5A). rBiFC generates a YFP signal when the N‐ and C‐terminal halves of YFP anneal to form a stable complex. Following *Agrobacterium* infiltrations to transform the leaves, we incubated plants in 400 μbar and 1,000 μbar CO_2_ for 48 h before confocal imaging. Ratiometric analysis of YFP fluorescence relative to the RFP signal (Figure 5B, C) showed that KAT1 interaction with SYP121 was reduced significantly in elevated CO_2_, although there was no effect on SYP121 binding with its cognate SNARE partner SNAP33 (Figure 5C). Immunoblot analysis verified the expression of split‐YFP tagged proteins (Figure 5D).

To confirm these findings, we carried out co‐immunoprecipitation experiments in Arabidopsis transiently transformed to express GFP‐KAT1. Following treatments in control and 1 mM HCO_3_^−^, GFP‐KAT1 immobilized on GFP‐trap resin as bait recovered co‐immunoprecipitant native SYP121, although SYP121 binding with the channel was significantly reduced in HCO_3_^−^‐treated leaf tissue (Figure 5E, F). As a negative control, we also tested SYP132, a close SNARE homolog that acts independently of the K^+^ channel (Xia et al., 2019). As expected, we did not observe any SYP132 bound to the bait. Taken together, these data suggest an uncoupling of the SNARE and channel with CO_2_ consistent with reduced SYP121‐mediated secretory traffic and a bias to the inactive K^+^ channel.

### SYP121 and CO_2_ coregulation of KAT1 affects stomatal function and plant growth

Stomatal closure depends on reduced plasma membrane H^+^‐ATPase activity, Cl^−^ anion efflux through SLAC1, and inhibition of K^+^ influx through KAT1 (Blatt, 2000; Jezek and Blatt, 2017; Zhang et al., 2018b). Conversely, stomata open when SLAC1 activity is suppressed and KAT1 activity is enhanced for K^+^ ion uptake into the guard cells (Eisenach et al., 2012; Jezek and Blatt, 2017). In control conditions, SYP121 promotes channel‐mediated K^+^ uptake during stomatal opening, both through secretory vesicle traffic and enhanced channel gating, and its impact extends to stomatal closing (Eisenach et al., 2012; Jezek and Blatt, 2017; Leyman et al., 1999). Thus, we reasoned that if CO_2_ action on KAT1 depends on SYP121, then stomatal conductances should be slowed with steps both above and below ambient CO_2_ in the absence of the SNARE.

**Figure 6:**
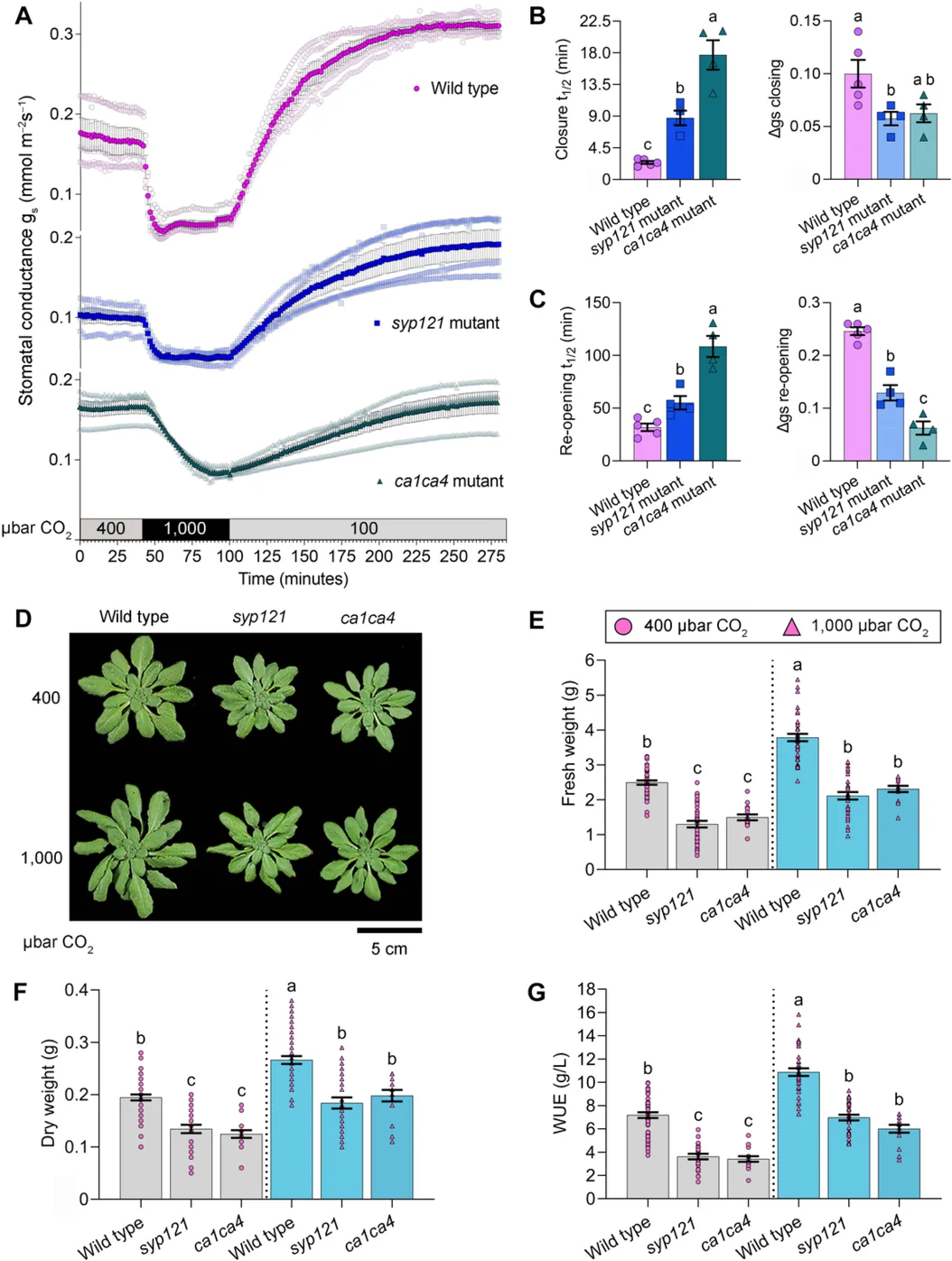
SYP121‐dependent CO_2_ regulation of KAT1 affects stomatal function and plant growth **(A)** Stomatal conductance (g_sw_) for wild‐type, *syp121*, and *ca1ca4* mutant Arabidopsis in response to steps between 400, 1,000, and 100 μbar CO_2_ (bottom). Data points are independent biological replicates (transparent colors, *n* ≥ 4 per genotype) and their means ± *SE* (solid colors). **(B**, **C)** Stomatal closing (400 to 1,000 μbar CO_2_) half‐times **(B)** and stomatal reopening (1,000 to 100 μbar CO_2_) half‐times **(C)**, derived from nonlinear least‐squares fittings to single‐exponential functions. Differences in the g_sw_ dynamic range (Δgs) are indicated (right) for closing and re‐opening. Data are means ± *SE* (*n* ≥ 4). Significant differences determined by Welch and Brown–Forsythe tests are indicated by letters (*P* < 0.05). **(D)** Representative wild‐type, *syp121* and *ca1ca4* mutant Arabidopsis after 4 weeks of growth under 400 μbar and 1,000 μbar CO_2_. Scale bar = 5 cm. **(E**–**G)** Fresh weight **(E)**, dry weight **(F)**, and water‐use efficiency (WUE) **(G)** derived for the plants in **(D)**. Data are means ± *SE*, shown as bars with data point scatter for each set of plants (*n* ≥ 15 plants). Significance with *P* values was assessed using the Brown–Forsythe and Welch ANOVA method (*P* < 0.05). Also see Figures S13, S14.

To test this hypothesis, we used *in vivo* infrared gas‐exchange analysis (IRGA) to measure the stomatal conductance of intact leaves from 4‐week‐old wild‐type and *syp121* mutant Arabidopsis. Additionally, we included *ca1ca4* mutant plants (Figure S7B, C) as a control for CO_2_ in equilibration with H_2_CO_3_ and dissociation to HCO_3_^−^ mediated by the carbonic anhydrases. All plants were pre‐adapted to 60% RH at 22.5°C and 200 μmol m^−2^ s^−1^ light and 400 μbar CO_2_, which is equivalent to the ambient concentration of the gas in air. As expected, no significant differences in transpiration and stomatal conductance (g_s_) were observed in the steady state between the wild‐type and *ca1ca4* mutant plants. Only the *syp121* mutant showed reduced g_s_ in the steady state (Figures 6A, S13A, B). With steps in CO_2_ to 1,000 and 100 μbar CO_2_, relaxations in g_s_ were greatly slowed in both the *ca1ca4* and *syp121* mutants (Figure 6A–C). Analysis of the stomatal conductance changes (Δg_s_) showed that both stomatal closure and reopening in response to CO_2_ steps were significantly reduced in the *syp121* and *ca1ca4* mutants relative to the wild‐type plants (Figure 6A–C), consistent with an overall inhibition of stomatal movement rather than simply a slowing of stomatal kinetics in response to CO_2_.

We analyzed vegetative plant growth, measuring shoot fresh and dry weights after 4‐week growth of the same plants under 400 and 1,000 μbar CO_2_. Normally, *syp121* mutant Arabidopsis show a growth phenotype only when grown under high light (200 μmol m^−2^ s^−1^ PAR) and low humidity (55% RH) (Eisenach et al., 2012). Therefore, we expected the growth of both *syp121* and *ca1ca4* mutant plants to phenocopy the wild type in elevated CO_2_. Remarkably, although the overall growth was higher under elevated CO_2_, both the *syp121* and *ca1ca4* mutants were smaller than wild‐type Arabidopsis. We calculated the plant water‐use efficiency (WUE) as the amount of dry biomass produced per unit water transpired. The results showed significant growth reductions in both the mutant lines relative to the wild type (Figure 6D–G).

Concurrent analysis of carbon assimilation rates (A) against internal CO_2_ (C_i_) indicated that photosynthetic efficiency was not a factor behind the reduced growth in the two mutants (Figure S13C). We also examined plants grown under 400 and 1,000 μbar CO_2_ for transcript and SYP121 protein abundance. qRT‐PCR analysis showed that *SYP121* expression in both wild‐type and *ca1ca4* mutant plants was reduced at ambient CO_2_, and even more, under elevated CO_2_ (Figure S14A; Table S1). Immunoblot analysis using anti‐SYP121 antibodies (Geelen et al., 2002; Tyrrell et al., 2007) showed that SYP121 expression was significantly reduced at the plasma membrane in *ca1ca4* mutants compared to wild‐type plants (Figure S14B, C). Significant overexpression of SYP121 promotes its aggregation in Arabidopsis (Grefen et al., 2010b); therefore, we tested gas exchange in *syp121* null mutant plants complemented to moderately overexpress the SNARE (SYP121‐OX) and found that stomatal conductance (g_s_) was comparable to the wild‐type Arabidopsis (Figure S14D, E). Overall, these results suggest that SYP121 contributes to plant growth through stomatal responsiveness to CO_2_, likely through CO_2_‐sensitive vesicle trafficking.

## DISCUSSION

Stomatal movements track the immediate demands of photosynthesis for CO_2_ while limiting transpirational water loss (Blatt, 2000; Meckel et al., 2007; Lawson and Blatt, 2014; Jezek and Blatt, 2017), and thus impact agricultural productivity and the carbon and water cycles across the globe (Ruiz‐Vera et al., 2013; Lawson and Blatt, 2014; Office, 2017). While there is substantial literature pertaining to CO_2_ regulation of ion transport and metabolism in stomatal guard cells, to date, very little information is available that speaks to the impacts of CO_2_ on membrane vesicle traffic, not least as it affects ion transporter populations relevant to guard cell function. We now show that traffic of the KAT1 K^+^ channel, which facilitates K^+^ uptake for stomatal opening, is subject to CO_2_ control through secretory traffic associated with the plasma membrane SNARE protein SYP121. To this end, we find (i) that increasing the atmospheric partial pressure of CO_2_ alters KAT1 mobility at the plasma membrane; (ii) that KAT1 mobility arises through a relocation of the channel protein to endomembrane compartments, thereby affecting channel abundance at the plasma membrane; and (iii) that CO_2_ action is subject to the SNARE protein SYP121, and impacts stomatal movements, plant growth, and water‐use efficiency. These findings uncover parallels to ABA‐evoked membrane vesicle traffic, in addition to highlighting some important differences. They extend our understanding of CO_2_ action in guard cell ion transport to encompass a regulation of transporter populations available at the cell membrane and they offer unprecedented evidence for vesicle trafficking in response to elevated CO_2_ in a plant cell system.

### Parallels to ABA‐evoked traffic of the KAT1 channel

Central to our discoveries was the finding that elevated HCO_3_^−^, equivalent to an increase in CO_2_ roughly to 1,000 μbar, promoted KAT1 internalization from the membrane. We observed mCherry‐KAT1 fluorescence at the cell periphery to decay with a half‐time of around 9 min when challenged with 1 mM HCO_3_^−^ (Figure 1). This behavior is similar to the time scales for endocytosis that we observed (Figure S3) and were reported previously (Sutter et al., 2007) on adding ABA. A limited kinetic analysis suggested an apparent *K*_1/2_ for HCO_3_^−^ action near 0.5 mM (Figure S2), a value that conforms with the range of CO_2_ partial pressures and, hence, of HCO_3_^−^ concentrations at which stomata normally respond (Farquhar, 1974; Mott, 1990).

In support of these observations, we note that the loss in fluorescence was parallelled by a proportional loss of KAT1 protein from the plasma membrane and gain in the inner membrane fraction from Arabidopsis leaves challenged with 1,000 μbar CO_2_, and with no apparent change in expression (Figure 2). Together, the findings point to a substantial re‐localization of the K^+^ channel away from the plasma membrane across a physiologically relevant range in CO_2_ partial pressures and HCO_3_^−^ concentrations in solution. These events occur over time scales that, like ABA‐evoked KAT1 internalization, are too slow to account for the changes in channel activity that have been resolved on elevating CO_2_ (Brearley et al., 1997). However, KAT1 endocytosis is consistent with adaptive behaviors to stress that depend on changes in the population of K^+^ channels at the guard cell plasma membrane (Lebaudy et al., 2008).

### A SNARE dependence to KAT1 mobilization at the plasma membrane

KAT1 internalization is also accompanied by an apparent mobilization of the channel more broadly within the plane of the membrane. Challenge with ABA was shown previously to lead to channel endocytosis from the focal points of KAT1 puncta (Sutter et al., 2007). With elevated CO_2_/HCO_3_^−^, however, we found that channel endocytosis progressed more widely from the plasma membrane, leading to an increase in RSD of the fluorescence signal at the cell surface (Figure 1B–D). It is as if the baseline in fluorophore detection had been increased, thereby reducing the overall signal, while at the same time masking the fluorescence signal between puncta. An increase in CO_2_/HCO_3_^−^ also resulted in a broadening of the mCherry‐KAT1 signal associated with each punctum (Figure 4A). Indeed, FRAP measurements showed a highly significant increase in mCherry‐KAT1 recovery following local photobleaching in tissues treated with 1 mM HCO_3_^−^ (Figure 1E, F). In the absence of changes in transcription (Figure 2J) and the immunochemical evidence for wider removal of the channel from the plasma membrane in response to HCO_3_^−^ (Figure 2), the only reasonable explanation is an increase in lateral mobility of channels within the plane of the membrane concurrent with endocytosis beyond the punctal limits.

Remarkably, we also found that KAT1 mobility with CO_2_/HCO_3_^−^ was reduced on co‐expressing the SNARE SYP121 (Figures 3A–D, S9). SYP121 was previously shown to be important for trafficking and recycling of the K^+^ channels as well as for stomatal responsiveness to water stress and ABA (Leyman et al., 2000; Geelen et al., 2002; Eisenach et al., 2012). In light of this background, it was surprising that KAT1 mobility should be reduced on overexpressing the SNARE. The findings gained further support from our observation (Figure 3E, F) that the closely related SNARE SYP132, which does not contribute to KAT1 traffic (Xia et al., 2019), had no effect on the mobility of the channel.

However, KAT1 also binds physically with SYP121, a binding that promotes vesicle traffic as well as K^+^ channel activity (Grefen et al., 2015; Lefoulon et al., 2018; Waghmare et al., 2019; Zhang et al., 2019). KAT1–SYP121 binding was evident, if reduced, on increasing CO_2_ (Figure 5). We suspect, therefore, that overexpressing the SNARE also stabilizes the channel at the membrane through their physical interaction. This conclusion accords with the loss of punctal expansion when challenged with HCO_3_^−^ (Figure 4B) and the stability of KAT1 in plasma membrane fractions (Figure 2). As a corollary, it also suggests that the regulation of KAT1 activity at the membrane through SYP121 binding is also closely integrated with subsequent events of channel endocytosis and sequestration (Sutter et al., 2006; Eisenach et al., 2012). It follows, too, that reduced binding of SYP121 may be predicted to facilitate KAT1 mobility and internalization from the membrane in a CO_2_‐sensitive manner. Whether or not this prediction proves correct, the findings to date underline a role for vesicle traffic, and especially the association of the KAT1 channel with SNARE SYP121, in regulating guard cell K^+^ transport over time scales from seconds to many tens of minutes in response to CO_2_.

### Implications for stomatal physiology and vegetative plant growth

One immediate consequence of interfering with SYP121 function *in vivo* was a substantial slowing of stomatal kinetics to changes in atmospheric CO_2_ partial pressure. Both stomatal opening and closing were affected in the *syp121* null mutant (Figure 6A–C). The impacts on CO_2_‐dependent kinetics contrast the effects of the mutant on manipulating [Ca^2+^]_i_, which was reported to slow stomatal reopening but with little impact on stomatal closing (Eisenach et al., 2012).

Stomatal movements are generally driven by the osmotically active transport of K^+^ in conjunction with that of Cl^−^ and malate (Mal) ions across the guard cell membrane (Blatt, 2000, 2024; Meckel et al., 2007; Sutter et al., 2007; Lawson and Blatt, 2014; Brodribb and McAdam, 2017; Jezek and Blatt, 2017). Closure is achieved principally with the efflux of these solutes and is, in large part, regulated by elevations in [Ca^2+^]_i_ that inactivate KAT1‐mediated transport and the H^+^‐ATPases while activating the anion currents carried by the SLAC1 and SLAH3 channels (Assmann and Jegla, 2016; Jezek and Blatt, 2017; Blatt, 2024). One immediate explanation for the differences in stomatal closing with CO_2_ and [Ca^2+^]_i_ is that SYP121 acts through a parallel pathway with elevated CO_2_. Thus, it is tempting to draw connections between the effects of the *syp121* and *ca1ca4* mutants, both of which affect opening as well as closing (Figure 6A–C). Indeed, we noted that SYP121 expression was reduced in the *ca1ca4* mutant (Figure S14A–C), suggesting a functional association between the two proteins. Such an interpretation would accord also with the reduced binding of SYP121 with KAT1 in elevated CO_2_ conditions (Figure 5). Regardless, slowing stomatal kinetics at the whole‐plant level has a demonstrable and profound impact on vegetative growth subject to atmospheric CO_2_ (Figure 6D–G), and the findings therefore place the SNARE and its juxtaposition with KAT1 squarely within the context of CO_2_‐dependent stomatal physiology.

**Figure 7:**
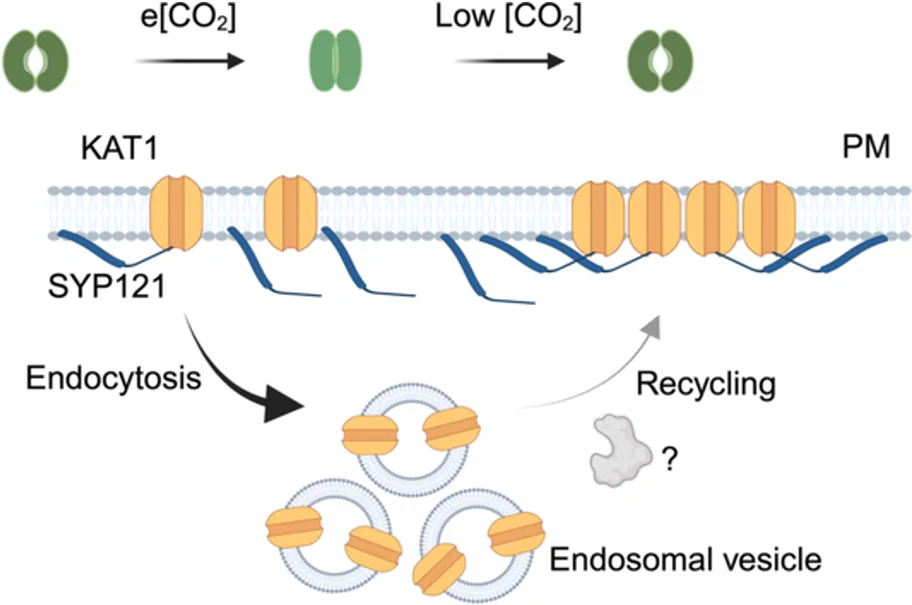
Schematic model illustrating CO_2_ regulation of KAT1 traffic dependent on SNARE SYP121 KAT1 endocytosis in elevated CO_2_ regulates channel abundance at the plasma membrane (PM). SYP121 binding stabilizes the channels in membrane microdomains, and, in juxtaposition with KAT1 trafficking, is a key factor for CO_2_‐sensitive changes in stomatal movements. As yet unknown factors contributing to channel recycling to the plasma membrane are depicted and warrant future studies. Created in BioRender.

In summary, we have uncovered CO_2_ regulation of the plant membrane vesicle trafficking machinery in plants that governs KAT1 channels, with the trafficking SNARE SYP121 as the central component (Figure 7). It can be expected too that additional as yet unidentified components contribute to CO_2_‐sensitive vesicle trafficking that future studies should focus on. Our findings highlight the importance of CO_2_‐regulated membrane traffic that affects stomatal gas exchange, plant growth, and water use, and provides a new perspective toward the understanding of plant behaviors in response to climate change.

## MATERIALS AND METHODS

### Plant material and plasmid construction

*Arabidopsis thaliana* ecotype Columbia‐0 (Col‐0) and wild‐type *Nicotiana tabacum* (Tobacco) plants were used as models in the study. Transgenic Arabidopsis plants used in the analysis were derived from KAT1 knockout mutant (*kat1*) seeds (Locascio et al., 2019) (SALK93506, carrying the T‐DNA insertion in the first exon), CA1/CA4 double mutant (*ca1ca4*) seeds (Hu et al., 2010) (NASC N66122) obtained from Nottingham Arabidopsis Stock Centre (NASC, http://arabidopsis.info/), SYP121 null mutant (*syp121‐1 = pen1*) seeds (Collins et al., 2003), and SYP121‐OX seeds, T2 generation (Grefen et al., 2010b) moderately overexpressing GFP‐tagged SYP121 driven by the pUBQ10 promoter in the *syp121* null mutant background.

Arabidopsis KAT1, SYP121, and SYP132 entry clones and pUBC‐GFP‐SYP121^DC^ (GFP‐SYP121Sp2) constructs were from studies (Karnik et al., 2013; Zhang et al., 2015). The open reading frame for *KAT1* was amplified to include a native stop codon and Gateway® cloning attachment sites (attB3/B2, or attB1/B4) to produce entry clones. All destination vectors for expression in plants were produced using Multisite Gateway® cloning (Karimi, 2002). For confocal analysis, the pFRETgc‐2in1‐NN vector (Hecker et al., 2015) was used to drive transient co‐expression of GFP‐ or mCherry‐fusion proteins, each under the control of the CaMV35S promoter. Protein binding studies were carried out using the ratiometric pBiFCt‐2in1‐NC and pBiFCt‐2in1‐CC vectors (Grefen and Blatt, 2012) for co‐expressing nYFP‐ and cYFP‐fusion proteins, and RFP as a ratiometric transformation marker under the CaMV35S promoter. For co‐immunoprecipitation analysis, Arabidopsis leaves that were transiently transformed to express GFP‐KAT1 under the CaMV35S promoter were used.

### Plant growth and transformation

Wild‐type tobacco (*Nicotiana tabacum*) plants were grown at 26°C on soil under 16 h‐light/8 h‐dark cycles, with 200 μmol m^−2^ s^−1^ PAR light and 70% restricted humidity. Using *Agrobacterium tumefaciens* GV3101 carrying the desired constructs (Clough and Bent, 1998), three to four fully expanded leaves were infiltrated for transient transformation of the leaf epidermis. Arabidopsis seedlings for imaging stomata were grown on 0.25× Murashige & Skoog (MS) media (pH 6.0) prior to transient transformation (Li et al., 2009).

For RT‐qPCR analysis, Arabidopsis seeds were germinated and grown on 1/2MS media for 2 weeks prior to separation of shoot and root tissue. For soil‐grown plants, seedlings were transplanted to 7‐cm pots for growth in CO_2_‐controlled GEN1,000 growth chambers (Conviron, Isleham, UK) using 8 h‐light/16 h‐dark, 22°C/18°C cycles under 150 μmol m^−2^ s^−1^ white light (total daily fluence of 4.2 mol m^−2^ PAR) and 400 or 1,000 μbar CO_2_ gas. Soil water content was monitored using an ML3 moisture sensor (DeltaT Devices, Cambridge, UK), and pots were watered daily to maintain 15% ± 2% soil water content. Plants were harvested at 4 weeks of growth, and fresh and dry weights were measured. Water‐use efficiency (WUE) was calculated as

$$\mathrm{WUE}=\mathrm{DW}\;\left(g\right)/\mathrm{Total}\;H_{2}O\;\mathrm{given}\;\left(L\right)$$

### CO_2_ and HCO_3_^−^ treatments

For confocal microscopy and HCO_3_^−^ analysis, solutions were prepared with and without added NaHCO_3_ salt and buffered in 5 mM MES with pH adjusted to 6.5 using NaOH as necessary. All solutions were prepared fresh and used within 30 min to infiltrate leaves. Note that H_2_CO_3_ conversion into CO_2_ in solution has a half‐time of around 1 h.

For CO_2_ gas treatments, plants were placed in CO_2_‐controlled growth chambers at ambient (400 μbar) and high (1,000 μbar) CO_2_ in air.

### RNA isolation and RT‐qPCR

Total RNA was extracted from plant tissue using the TRIzol™ Plus RNA Purification Kit (Invitrogen, Paisley, UK) following the manufacturer's instructions. One microgram of RNA was used for cDNA synthesis with the QuantiTect Reverse Transcription Kit (Qiagen, Manchester, UK). RT‐qPCR was performed using Brilliant III Ultra‐Fast SYBR Green Master Mix (Agilent, Santa Clara, USA) on a StepOnePlus Real‐Time PCR System (Applied Biosystems, Thermo Fisher Scientific, UK). To determine expressions of *KAT1*, *KAT2*, *HAK5*, *SYP121*, and *βCA4* transcripts relative to the mitochondrial 18S rRNA (AtMg01390) reference gene, RT‐qPCR was carried out with gene‐specific primers (see Table S1 for primer sequences), calculated as 2^−ΔCt^, and fold changes in transcripts ± treatments were determined using the 2^−ΔΔCt^ method (Livak and Schmittgen, 2001).

### Confocal microscopy

Confocal images of tobacco leaf lower epidermal cells 2–3 d post infiltration were collected using a Leica TCS SP8‐SMD confocal microscope with spectral GaAsP detectors using 20×/0.85 NA dry and 40×/1.3 NA oil lenses (Leica, Wetzlar, Germany). GFP fluorescence was collected over 495 to 530 nm following excitation at 488 nm. mCherry fluorescence was determined over 595 to 630 nm following excitation at 552 nm. RFP was excited using the 543‐nm laser line, and emission was determined over 560 to 615 nm. YFP was excited with 514‐nm light, and YFP fluorescence emission was determined over 521 to 553 nm. Image analysis was carried out using ImageJ/Fiji (National Institutes of Health) software. In parallel, protein expressions were verified by immunoblot.

### Quantification of fluorescence distribution

Fluorescence was measured from images acquired at 0, 10, 15, 20, and 30 min following treatments across a region of interest (ROI) traced as a line (~1.5 μm wide) along the cell periphery using a bright‐field image overlay as the reference (Eisenach et al., 2012; Xia et al., 2019). The mean relative fluorescence values from different ROI were plotted against time and fluorescence decay half‐times were calculated using nonlinear least‐squares fitting.

### Fluorescence distribution at the cell periphery for puncta

To quantify the proportion of punctate fluorophore‐tagged protein distributions, percentage relative *SD* analysis was used as described before by (Xia et al., 2019). A percentage relative *SD* of intensities was determined from a one pixel‐wide line demarcating the cell periphery and normalized to the intensity means.

### Puncta diameter distributions

Punctal diameters were measured from equal numbers of randomly chosen puncta for each sample using confocal images acquired along the top of the cells. Frequency histograms of puncta diameters were fitted to Gaussian functions using a Levenberg–Marquardt algorithm (Marquardt, 1963) to extract fluorescence peak and full width at half‐maximum intensity (FWHM) dimensions.

### Fluorescence recovery after the photobleaching (FRAP) assay

Following treatments, five image scans were acquired for each prebleach baseline prior to the mCherry fluorophore photo bleach routine using a 100% intensity 552‐nm laser. Postbleach images were acquired every 4 s, over 180 s. A region of interest along the cell periphery marked by the bright‐field image overlay was chosen and fluorescence intensity was measured over time from the image stack. Fluorescence measurements were normalized to prebleach values to track fluorescence signal recovery over time. The best‐fit curve was generated using non‐linear least‐squares single‐exponential association and half‐times (*t*_1/2_) were calculated from the curve fittings.

Kymographs that track the fluorescence signal across each ROI averaging over a width of 9 pixels (~4.6 μm) within the photobleached area along the edge of a cell were obtained to visualize changes in fluorescence over time, with data from pre‐bleach images at −5 s, followed by post‐bleach images up to 185 s. Fluorescence intensity was color‐coded using the Fiji 16‐color panel, with a scale representing the fluorescence ratio from 0 to 1 for each ROI along the horizontal axis; the vertical axis showed time progression.

### Ratiometric bimolecular fluorescence complementation (rBiFC) assay

Tobacco plants were grown under ambient or elevated CO_2_ (1,000 μbar) for least 2 h before and for 2 d following *Agrobacterium* infiltration for transient expression of the rBiFC constructs (Grefen and Blatt, 2012). YFP and RFP fluorescence intensities in the images were determined using ImageJ software and ratiometric fluorescence analysis, and the YFP/RFP ratio was used to evaluate the rBiFc signal relative to the expression marker (RFP).

### Membrane fractionation

Microsomal total membranes extracted from leaf tissue were fractionated into enriched plasma and internal membranes as before (Waghmare et al., 2024) with the Minute Plant Plasma Membrane Protein Isolation Kit (Invent Biotech, Plymouth, USA) following the manufacturer's instructions. The protocol uses differential centrifugation to yield highly pure plasma membrane and internal membranes. Following protein estimation using the Bradford assay (Bradford, 1976), membrane fractions at equal total protein in control and treated samples each were resuspended in 2× Laemmli protein sample buffer (Bio‐Rad, UK) prior to separation using SDS‐PAGE and immunoblot analysis. Internal membrane fraction samples were prepared at 20‐fold higher total protein to ensure that proteins that occur predominantly at the plasma membrane are detected (as before (Baena et al., 2022, 2024)).

For co‐immunoprecipitation analysis, total microsomal fractions were isolated as described previously (Xia et al., 2019) from freshly harvested Arabidopsis leaf tissue from seedlings that were washed and incubated in water as a control and with 1 mM bicarbonate as indicated above. Microsomal membranes were collected by centrifuging supernatants at 100,000 *g* at 4°C for 1 h and resuspended in 5 mM phosphate buffer, pH 7.8, 330 mM sucrose, 0.1 mM EGTA, 0.1 mM EDTA‐KOH, 1 mM DTT, and protease inhibitor cocktail prior to dilution with the binding buffer (PBS, 0.01% v/v TritonX‐100, 0.05 mM DTT, 0.01% CHAPS, protease inhibitor) and incubated with GFP‐Trap resin (Chromotek, Munich, Germany) for 1 h at 4°C with gentle mixing. The resin was washed 3–4 times with binding buffer containing additional 150 mM NaCl, and GFP‐tagged bait and its co‐immunoprecipitant proteins were eluted with Elution buffer (50 mM Tris–HCl, pH 6.8, 5% w/v SDS, 2 mM EDTA, 0.1% v/v Triton X‐100, 50 mM DTT, 12% v/v glycerol, 0.05% w/v bromophenol blue) for subsequent analysis with immunoblotting.

### Immunoblotting

Sample preparation and immunoblot analysis of leaf tissue were carried out as described before (Karnik et al. 2015; Waghmare et al., 2024). Briefly, protein samples were resuspended in Laemmli buffer prior to separation using SDS‐PAGE, and separated proteins were transferred to nitrocellulose membranes (Bio‐Rad, UK). Immunoblotting was performed using protein‐specific antibodies that were commercially available or custom‐made (Eurogentech, Seraing, Belgium).

Custom‐made polyclonal antibodies against *Arabidopsis thaliana* proteins were raised in rabbits following immunization with KLH‐conjugated antigenic peptides containing unique epitopes: synthetic [βCA4 peptide NKIKEEHKDLSYDDQ] and affinity‐purified [SLAC1 N‐peptide residues 1–189, 21.6 kDa, (Xia et al., 2026)] as antigens and verified before use. The antibodies were applied as α‐KAT1 [1:3,000 dilution; Agrisera, Vännäs, Sweden (Xia et al., 2019)], α‐βCA4 (this study, 1:5,000 dilution; Eurogentec, Seraing, Belgium), α‐SYP121 (1:40,000 dilution; Agrisera (Tyrrell et al., 2007)), α‐SLAC1 (this study, 1:1,000 dilution), α‐SYP132 ((Xia et al., 2019); this study, 1:1,000 dilution), αmCherry (1:5,000 dilution; Abcam, Cambridge, UK,), α‐GFP (1:1,000 dilution; Abcam), α‐nYFP (1:5,000 dilution; Agrisera), α‐RFP (1:5,000 dilution; Abcam), and α‐myc (1:5,000 dilution; Chromotek, Planegg, Germany). Secondary antibodies used were horseradish peroxidase‐conjugated goat αrabbit (1:20,000 dilution; Abcam, ab6721) or goat α‐mouse IgG H&L (1:2,000 dilution; Abcam, ab6789). In some experiments, membranes were stripped in 25 mM glycine‐HCl, pH 2.2, supplemented with 1% SDS for 60 min with continued agitation before re‐probing with antibodies. Cross‐reacting bands in immunoblots were visualized using SuperSignal West Femto Maximum Sensitivity Substrate detection (Thermo Fisher Scientific, UK) and imaged by the Fusion FX Chemiluminescence imager (Vilber, France). Immunoblot band intensities in each lane for different proteins were measured using ImageJ (Fiji) and normalized against total protein in each lane detected with Coomassie staining.

### Time‐resolved stomatal conductance measurement

Intact leaves from 4–5‐week‐old Arabidopsis plants were clamped for time‐resolved stomatal conductance (gs) measurements using LiCOR 6800 IRGA systems (Lincoln, USA) with a leaf chamber. For CO_2_‐dependent stomatal conductance measurements, the clamped leaves attached to whole plants were equilibrated and stabilized at 200 μmol m^−2^ s^−1^ light intensity (6800‐01 A LED light source) at 60% relative humidity, 22.5°C, and 400 μbar CO_2_ for 1 h. For the stomatal reopening assay, leaves were kept at 400 μbar CO_2_ for 40 min as a control, followed by 1 h at 1,000 μbar CO_2_ and then 3 h at 100 μbar CO_2_. To minimize biological variation, wild‐type and mutant plants were grown side by side and analyzed within the same time period. Additionally, gas exchange experiments were repeated independently at various times of the year. All plants were examined at the same time relative to the diurnal cycle on at least 3 different days, and the measurements were normalized for leaf area using ImageJ.

### Statistical analysis

Statistical analyses were performed using GraphPad Prism version 10.4.2. Data are expressed as means ± *SE* of *n* observations. Depending on the data set, significance was determined using the Mann–Whitney test, or one‐ or two‐way ANOVA with appropriate post hoc comparisons (Tukey's, Holm‐Šídák or Fisher's LSD test, *P* < 0.05). For non‐normally distributed data or unequal variances, Brown–Forsythe and Welch ANOVA or Kruskal–Wallis tests were applied. Statistical significance was indicated at *P* < 0.05 unless otherwise noted.

### Accession numbers

Sequence data from this work can be found in the Arabidopsis Genome Initiative or GenBank/EMBL databases under the following accession numbers: *KAT1* (At5g46240.1), *KAT2* (At4g18290.2), *AHA1* (At2g18960.1), *βCA1* (At3g01500.2), *βCA4* (At1g70410.2), *SYP121* (At3g11820.1), *SYP122* (At3g52400.1), and *SYP132* (At5g08080.2).

## CONFLICTS OF INTEREST

The authors declare no conflicts of interest.

## AUTHOR CONTRIBUTIONS

R.K. and M.R.B. conceived the project. R.K. designed the experiments. R.K. and S.W. supervised the work. S.W. optimized biochemistry assays. S.W. and Z.Y. performed binding assays with support from S.F. for immunoblotting and membrane fractionation work. Z.Y. conducted experimental work, including imaging and physiology experiments. Z.Y., S.W., R.K., and M.R.B. analyzed the data. R.K. and M.R.B. wrote the manuscript. All authors have read and approved the contents of this paper.

## Supporting information

Additional Supporting Information may be found online in the supporting information tab for this article: http://onlinelibrary.wiley.com/doi/10.1111/jipb.70269/suppinfo

**Figure S1.** *KAT1* gene expression in different tissue types
**Figure S2.** CO_2_ regulation of KAT1 fluorescence density
**Figure S3.** CO_2_‐ and ABA‐sensitive KAT1 trafficking are kinetically similar
**Figure S4.** CO_2_ regulation of KAT1 distribution in Arabidopsis guard cells
**Figure S5.** Validation of FRAP assays
**Figure S6.** CO_2_‐sensitive mCherry‐KAT1 redistribution reduces channel density at the plasma membrane
**Figure S7.** Verification of antibodies against native Arabidopsis KAT1 and βCA4 proteins
**Figure S8.** Expression levels of *KAT1*, *KAT1*, and *HAK5* potassium channels
**Figure S9.** SNARE limits KAT1 mobilization at the plasma membrane in CO_2_
**Figure S10.** Co‐expression of SYP121 inhibits HCO_3_^−^‐sensitive mCherry‐KAT1 redistribution from the plasma membrane
**Figure S11.** SYP121 influences ABA‐sensitive KAT1 channel kinetics
**Figure S12.** Bright‐field overlay images and immunoblots verifying protein expressions in tobacco
**Figure S13.** CO_2_ regulation of KAT1 traffic affects stomatal function
**Figure S14.** *SYP121* gene expression and protein distribution in CO_2_
**Table S1.** RT‐qPCR reactions were performed using gene‐specific primers

## Data Availability

Vectors are available at https://plantscienceglasgow.org/facilities/.
