# Supplementary material for: CO2‐sensitive K+ channel traffic affects stomata and whole‐plant water use
Source: J Integr Plant Biol. 2026 Apr 22;68(9):3391–408. doi: 10.1111/jipb.70269 (PMC13545853; doi:10.1111/jipb.70269)
Supplement: Supplementary file 1 [file JIPB-68-3391-s001.docx]

**SUPPLEMENTAL DATA**

**Figure S1. KAT1 gene expression in different tissue types.**

**(A)** RT-qPCR analysis is shown that mitochondrial 18s rRNA (AtMg01390) expression as internal reference on separated shoots (leaves) and roots from two-week-old wild type Arabidopsis plants or leaf epidermis peels (enriched guard cells) from three-week-old wild type Arabidopsis (Pantaleno *et al.*, 2024; Weyers and Travis, 1981). Fold change of KAT1 expression (2^−ΔCt^) in different tissues. Data are mean ± *SE*, representative of four biological replicates. Statistically significant differences are indicated with P value using one-way ANOVA Tukey’s multiple comparisons test (*P* < 0.05). **(B)** The 3D surface plot vertical view on the top of a single mCherry-KAT1 puncta. Relative mCherry-KAT1 fluorescence intensity within a range from 0 to 240 depicted using pseudo colour (scale on right panel).

**Figure S2. CO_2_-regulation of KAT1 fluorescence density.**

**(A)** Representative confocal images of *Nicotiana tabacum* epidermal cells expressing mCherry-KAT1, acquired on a single focal plane at 0, 10, 15, 20, 30-minute intervals, following treatment with water (control). Images show mCherry-KAT1 fluorescence (top panels) with region of interest (ROI) (white dashed line), zoomed ROI images (middle panels), and overlay showing bright-field, and chlorophyll fluorescence (bottom panels). Scale bar = 50 µm (Figure 1B). Bottom panels show mCherry-KAT1 fluorescence with ROI for HCO_3_^−^ treated cells. **(B)** Representative confocal images of *Nicotiana tabacum* epidermal cells expressing mCherry-KAT1 (left panels), acquired on a single focal plane at 15 min, following treatment with water (control), 0.1 mM HCO_3_^−^, 1 mM HCO_3_^−^ and 3 mM HCO_3_^−^. Images show mCherry-KAT1 fluorescence (top panels), overlay showing bright-field and chlorophyll fluorescence (bottom panels), *n* = 3. Scale bar = 50 µm. **(C)** Graph, [HCO_3_^−^] dose dependent steady-state fluorescence complement derived from image analysis, see **(B)**. Data was fitted to a hyperbolic function and yielded a dependence for HCO_3_^−^ action with an apparent K_1/2_ at 0.48 ± 0.03, *n* = 3.

**Figure S3. CO_2_ and ABA sensitive KAT1 trafficking are kinetically similar.**

**(A)** Representative confocal images of *Nicotiana tabacum* epidermal cells expressing mCherry-KAT1, acquired on a single focal plane at 0, 10, 15, 20, 30-minute intervals, following treatment with 20 µM ABA [(+) ABA] diluted in 0.02% (v/v) ethanol. Images show mCherry-KAT1 fluorescence (top panels) with region of interest (ROI) (white dashed line), zoomed ROI images (middle panels), and overlay showing bright-field, and chlorophyll fluorescence (bottom panels). Scale bar = 50 µm. **(B)** Plot of mean ± *SE* mCherry-KAT1 fluorescence measured along the cell periphery from images, see **(A)** and Figure 1, relative to the control (0 min). Fluorescence decay, halftime t_1/2_ = 8.7 ± 1.4 min, following 20 µM ABA treatment calculated using nonlinear least-squares fit as single exponential decay, *n* = 23. **(C)** Mean %Relative Standard Deviation (RSD) ± *SE* of mCherry-KAT1 fluorescence distribution 30 min after control or ABA treatments, measured across a 1.5-pixel-wide line along the periphery of cells. Statistical significance (*P* < 0.05) using Mann-Whitney test was determined for cumulative distributions from *n* =12 datasets. *P* value is indicated.

**Figure S4. CO_2_ regulation of KAT1 distribution in Arabidopsis guard cells.**

**(A**, **B)** Representative confocal images of *Arabidopsis thaliana* epidermis transformed to express mCherry-KAT1, acquired on a single focal plane at 30 min following treatment with water (control, left panels) or 1mM HCO_3_^−^ (right panels) in a wider field **(A)**, and single stoma **(B)**. Images are mCherry-KAT1 fluorescence (top panels), overlay showing bright-field and chlorophyll fluorescence (bottom panels). Scale bar = 5 µm. **(C)** Mean %Relative Standard Deviation (%RSD) ± *SE* mCherry-KAT1 fluorescence distribution 30 min after control or 1 mM HCO_3_^−^ treatments, see **(A)** measured across a 1.5-pixel-wide line along the periphery of cells from ≥ 3 image sets each. Statistical analysis (*P* < 0.05) comparing cumulative distributions using Mann-Whitney test, *n* = 12 datasets, *P* values are indicated.

**Figure S5. Validation of FRAP assays.**

**(A**, **C)** Kymographs of mCherry-KAT1 fluorescence (pseudo coloured) in selected microdomains (2 µm × 150 µm ROI) along the cell periphery were plotted over time. The dotted lines in the inset image correspond to the photo-bleached region (a) and an adjacent control region (b) that is distant from the FRAP area. Images are representative for control **(A)** and HCO_3_^−^ treated **(C)** tissue. Scale bar = 50 µm. Fluorescence intensity was plotted relative to the pre-bleach levels showing the recovery of mCherry-KAT1 signal post-bleach under control conditions **(B)** and after treatment with 1 mM HCO_3_^−^ **(D)** over time (seconds).

**Figure S6.** **CO_2_-sensitive mCherry-KAT1 redistribution reduces channel density at the plasma membrane.**

**(A)** Representative immunoblots detecting mCherry-KAT1 (110.7 kDa) (top panels), ER resident BiP (73.5 kDa) (middle panel). Bottom panel is Coomassie stained membrane for total protein in each lane. Total microsomal membrane (T), internal membrane (IM) and plasma membrane (PM) fractions were purified from *N. tabacum* leaf epidermal cells expressing mCherry-KAT1 following incubation without [control (−)] or with (+) 1 mM HCO_3_^−^ (30 min). Equal amount of protein was loaded per lane for each T, IM and PM fractions to compare −/+ HCO_3_^−^ treated samples. *Note.* IM fractions had ~20-fold more total protein per lane compared to PM samples. Purity of PM fractions was evaluated using BiP band density relative to IM as 96.8% ± 0.3%. **(B)** Mean ± *SE* mCherry-KAT1 density in immunoblots, see **(A)** relative to PM control. *n* = 6 independent experiments. Band intensities were normalised to total protein in each lane detected using Coomassie stain. Statistical significance was determined by unpaired t test using Holm-Šídák method (*P* < 0.05). *P* values are indicated. *Note*. Total protein in each lane was used as reference for quantification instead of any single protein for accuracy. Approximately two-fold decrease in mCherry-KAT1 density within plasma membrane fractions was noted in samples with HCO_3_^−^. Conversely, KAT1 density in inner membranes was significantly higher.

**Figure S7. Verification of antibodies against native Arabidopsis KAT1 and** **βCA4 proteins**.

**(A)** Immunoblots to evaluate specificity of the KAT1 antibody (Xia *et al.*, 2019). Purified plasma membrane and internal membrane fractions from Arabidopsis wild-type and *kat1* mutant from Arabidopsis leaf lysates were resolved on SDS-PAGE and treated with the α-KAT1 antibody in immunoblot assays (see Methods). Native Arabidopsis KAT1 protein (78.3 kDa) was detected in wild type, but not in *kat1* mutant plant lysates. Black lines (left) indicate the positions of molecular mass markers, and black arrows (right) indicate KAT1 position. **(B**, **C)** Immunoblots of Arabidopsis wild type and double mutant *ca1ca4* line with total proteins. Bands were resolved for **(B)** βCA1 (29.5 kDa) and **(C)** βCA4 (30.8 kDa). Black lines (left) indicate positions of molecular mass markers, and black arrows (right) indicate expected band positions, non-specific band (ns) is indicated. Total protein in each lane was detected using Coomassie blue staining of the membrane (right panels). Note. *ca1ca4* mutant plants lack expression of both βCA1 and βCA4.

**Figure S8. Expression levels of *KAT1*, *KAT1* and *HAK5* potassium channels.**

RT-qPCR analysis of mitochondrial 18S rRNA (AtMg01390) expression is shown as an internal reference of Arabidopsis leaves from 3–4 weeks-old wild type, *kat1*, and *syp121* mutant plants. The fold changes for *KAT1*, *KAT2* and *HAK5* expressions (2^−ΔCt^) in various sample types are shown. Data represents the means ± *SE* and are based on four biological replicates. Statistical significance was determined using two-way ANOVA with Tukey’s multiple comparisons test (*P* < 0.05), *n* = 3.

**Figure S9. SNARE limits KAT1 mobilisation at the plasma membrane in CO_2_.**

**(A)** Representative confocal images of *Nicotiana tabacum* epidermal cells co-expressing mCherry-KAT1 and GFP-SYP121, acquired on a single focal plane at 0, 10, 15, 20, 30-minute intervals, following treatment with water (control) or 1 mM HCO_3_^−^ [(+) HCO_3_^−^]. Images show fluorescence mCherry-KAT1 zoomed ROI images overlay with bright-field and chlorophyll fluorescence at 0, 10, 15, 20, 30 min intervals with top panels for control and bottom panels for 1 mM HCO_3_^−^. **(B)** Mean ± *S.E* mCherry-KAT1 fluorescence measured along the cell periphery from images (see Figure 5A), relative to the control (0 min), *n* ≥ 14. **(C)** Representative confocal images of *Nicotiana tabacum* epidermal cells co-expressing mCherry-KAT1 and GFP-SYP121, acquired on a single focal plane at 0, 10, 15, 20, 30-minute intervals, following treatment with water (control) or 1 mM HCO_3_^−^ [(+) HCO_3_^−^]. Images show mCherry-KAT1 with ROI (white dashed line) and GFP-SYP121 fluorescence in widefield at 0, 10, 15, 20, 30 min intervals with top panels for control and bottom panels for 1 mM HCO_3_^−^.

**Figure S10. Co-expression of SYP121 inhibits HCO_3_^−^ sensitive mCherry-KAT1 redistribution from the plasma membrane.**

**(A)** Immunoblots represent proteins mCherry-KAT1 (110.7 kDa) (first panel), GFP-SYP121 (66.5 kDa, second panel), ER resident BiP (73.5 kDa, third panel), and Coomassie stained membrane for total protein (fourth panel). Total microsomal membrane (T), internal membrane (IM) and plasma membrane (PM) fractions were purified from *N. tabacum* leaf epidermal cells co-expressing mCherry-KAT1 and GFP-SYP121 in control or (+) HCO_3_^−^ (30 min). Purity of PM fractions using BiP band density relative to IM was 94.8% ± 0.2%, *n* = 3. **(B)** The plot of mean ± *SE* mCherry-KAT1 density in immunoblot assays relative to total protein, see **(A)**. Statistical significance was determined using two-way ANOVA with Tukey’s multiple comparisons test (*P* < 0.05), *n* = 3. **(C)** The plot of mean ± *SE* GFP-SYP121 density in immunoblot assays relative to total protein, see **(A)**. Multiple unpaired t-test with Holm-Šídák’s multiple comparisons was used to determine statistical significance (*P* < 0.05), *n* = 3.

**Figure S11. SYP121 influences ABA-sensitive KAT1 channel kinetics.**

**(A)** Schematic diagram showing truncated SYP121 fragments. The structure of full length SYP121 (Methionine 1–Arginine 346) consists of an N-terminus with three subdomains (N), a Habc domain (Habc), a Qa-SNARE motif (Qa) and a C-terminal transmembrane domain (TM). The deletion mutant of SYP121-Sp2 (Methionine 1–Cysteine 288) is shown in the second panel. **(B**–**D)** Kymographic analysis of mCherry-KAT1 fluorescence intensity (pseudo coloured, see scale) plotted against time (seconds) under 20 µM ABA treatment at 30 min. Measurements were from microdomains (2 µm × 150 µm ROI, see inset dotted line along the periphery of epidermal cells co-expressing GFP **(**control, **B)**, GFP-SYP121 **(C)** or GFP-Sp2 fragment **(D)**. Representative confocal images are shown. Scale bar = 50 µm. **(E)** Mean ± *SE* mCherry-KAT1 fluorescence signal recovery post-bleach relative to pre-bleach fluorescence in 20 µM ABA treated cells co-expressing GFP (circles), GFP-SYP121 (triangles) or GFP-Sp2 fragment (squares). Plots are from ≥ 5 datasets fitted using nonlinear least-squares as a single-exponential association (bold lines). Fluorescence recovery half-times (t_1/2_) were calculated as 18.1 ± 8.8 s (GFP) and 38.5 ± 7.2 s (GFP-SYP121) and 24.8 ± 2.39 s (GFP-Sp2). No statistical significance was noted using Mann-Whitney test (*P* < 0.05), *n* = 3.

**Figure S12. Brightfield overlay images and immunoblots verifying protein expressions in Tobacco.**

**(A)** Confocal images (representative) acquired along a single plane at the cell surface in *N. tabacum* epidermis co-expressing mCherry-KAT1 with GFP (first panel), GFP-SYP121 (second panel), or GFP-SYP132 (third panel). Images show mCherry-KAT1 fluorescence following control (left panels) or 1 mM HCO_3_^−^ treatment (30 min, right panels) in widefield images overlay with bright-field and chlorophyll fluorescence. Scale bar =50 µm, (see Figure 4). **(B)** Immunoblots to verify expression of the KAT1 co-expressing with GFP, GFP-SYP121 and GFP-SYP132 with anti-mCherry (top panels) and anti-GFP antibodies (middle panels), respectively. Bands were resolved for GFP (27 kDa), GFP-SYP121 (66.5 kDa), GFP-SYP132 (63 kDa) and mCherry-KAT1 (110.7 kDa). Black lines (left) indicate positions of molecular mass markers, and black arrows (right) indicate expected band positions. Total proteins were stained with Coomassie blue (bottom panels).

**Figure S13. CO_2_ regulation of KAT1 traffic affects stomatal function.**

**(A)** Time-resolved intact leaf stomatal conductance in wild-type (circles), *syp121* (squares), and *ca1ca4* mutants (triangles) plants with CO_2_ shifts indicated at the top. Data points are shown mean ± *SE*, see Figure 6A. **(B)** Mean ± *SE* steady state stomatal conductance (gsw) at 400 µbar CO_2_. Statistical significance using one-way ANOVA with Tukey’s multiple comparisons test is shown using alphabets (*P* < 0.05), *n* = 4. **(C)** Assimilation rates as a function of the calculated CO_2_ inside the leaf (Ci) for wild-type (circles), *syp121* (squares), and *ca1ca4* mutants (triangles) plants under saturating photosynthetically active radiation (PAR, 600 µmol m^−2^ s^−1^). Data are means ± *SE* (*n* ≥ 3), with single curve using second order polynomial least squares fit (*P* = 0.43).

**Figure S14. SYP121 gene expression and protein distribution in CO_2_.**

**(A)** RT-qPCR analysis of mitochondrial 18S rRNA (AtMg01390) expression is shown as an internal reference of Arabidopsis plant leaves from 3–4 weeks-old wild type and *ca1ac4* mutant plants under 400 µbar CO_2_ (grey bars) and 1,000 µbar CO_2_ (black bars), respectively. The fold changes for SYP121 expression (2^−ΔCt^) in wild type and *ca1ac4* mutant plants are shown. Data represents the mean ± *SE* and are based on three biological replicates. Statistical significance was determined using two-way ANOVA (*P* < 0.05), *n* = 3. **(B)** Immunoblots of Arabidopsis wild type and *ca1ca4* double mutant plants with purified plasma membrane proteins under 400 µbar and 1,000 µbar CO_2_, respectively. Bands were resolved for SYP121 detecting by anti-SYP121 (38 kDa). Black lines (left) indicate positions of molecular mass markers, and black arrows (right) indicate expected band positions. Total proteins were stained with Coomassie blue (bottom panels). **(C)** The plot of mean ± *SE* SYP121 density in immunoblot assays relative to wild type under 400 µbar CO_2_, see **(B)**. Statistical significance depicted by alphabets was determined using two-way ANOVA with Fisher’s LSD test (*P* < 0.05), *n* = 4. **(D**–**E)** Mean halftimes ± *SE* of stomatal closing (step. 400 to 1,000 µbar CO_2_) **(D)**, and stomatal reopening (step. 1,000 to 100 µbar CO_2_) halftimes **(E)**, derived from nonlinear least-squares fittings to single-exponential functions. IRGA-based gas exchange analysis of stomatal conductance was in four-week-old intact leaves of Arabidopsis moderately over-expressing GFP-tagged SYP121 (SYP121-OX) in *syp121* mutant background in response to steps between 400, 1,000 and 100 µbar CO_2_ compared against wild-type and *syp121* mutant plants (Figure 6). Significant differences determined by Brown-Forsythe and Welch ANOVA (*P* < 0.05) are indicated by letters, (*n* ≥ 4).

**Table S1. RT-qPCR reactions were performed using gene-specific primers**

| **Gene name** | **Gene ID** | **Primer pair (5’-3’)** | **References** |
| --- | --- | --- | --- |
| *CA4.2* | At1g73400 | TGTCAGAAACATAGCCAATA GAGTCCCTTAATACCACCA | This paper |
| *SYP121* | At3g11820 | CACTGGAGGAAGTCAACC  TCACAAAAACCAAATAAACG | (Honsbein *et al.*, 2009) |
| *KAT1* | At5g46240 | GTGTCAGGAGCAGTGGACTT CATGCATCGCACTCATCAGC | This paper |
| *KAT2* | At4g18290 | TGCTCAACAGCTCCATTCCA  ACTGCAAACAGTGTCACCGA | This paper |
| *HAK5* | At4g13420 | AAGAGGAACCAAATGCTGAGACA GCCCCGATGAAGGGACAT | (Nieves-Cordones *et al.*, 2010) |
| *18S* | AtMg01390 | GATGAGCCTGCGTAGTATTAG AGTCATTCCGAAGAACACTTGC | (Armengot *et al.*, 2016) |

**REFERENCES**

**Armengot L, Caldarella E, Marques-Bueno MM, Martinez MC**. 2016. The Protein Kinase CK2 Mediates Cross-Talk between Auxin- and Salicylic Acid-Signaling Pathways in the Regulation of PINOID Transcription. PLoS One **11**, e0157168.

**Honsbein A, Sokolovski S, Grefen C, Campanoni P, Pratelli R, Paneque M, Chen Z, Johansson I, Blatt MR**. 2009. A tripartite SNARE-K+ channel complex mediates in channel-dependent K+ nutrition in Arabidopsis. Plant Cell **21**, 2859-2877.

**Nieves-Cordones M, Alemán F, Martínez V, Rubio F**. 2010. The *Arabidopsis thaliana* HAK5 K^+^ transporter is required for plant growth and K^+^ acquisition from low K^+^ solutions under saline conditions. Molecular Plant **3**, 326-333.

**Pantaleno R, Schiel P, García-Mata C, Scuffi D**. 2024. Analysis of Guard Cell Readouts Using *Arabidopsis thaliana* Isolated Epidermal Peels. Bio Protoc **14**, e5033.

**Weyers JDB, Travis AJ**. 1981. Selection and Preparation of Leaf Epidermis for Experiments on Stomatal Physiology. Journal of Experimental Botany **32**, 837-850.

**Xia L, Mar Marquès-Bueno M, Bruce CG, Karnik R**. 2019. Unusual Roles of Secretory SNARE SYP132 in Plasma Membrane H^+^-ATPase Traffic and Vegetative Plant Growth. Plant Physiology **180**, 837-858.
